# Assessing the role of evolutionary information for enhancing protein language model embeddings

**DOI:** 10.1038/s41598-024-71783-8

**Published:** 2024-09-05

**Authors:** Kyra Erckert, Burkhard Rost

**Affiliations:** 1 TUM School of Computation, Information and Technology, Bioinformatics & Computational Biology - i12, Boltzmannstr. 3, 85748 Garching/Munich, Germany; 2 TUM Graduate School, Center of Doctoral Studies in Informatics and Its Applications (CeDoSIA), Boltzmannstr. 11, 85748 Garching, Germany; 3 Institute for Advanced Study (TUM-IAS), Lichtenbergstr. 2a, 85748 Garching/Munich, Germany; 4 TUM School of Life Sciences Weihenstephan (TUM-WZW), Alte Akademie 8, Freising, Germany

**Keywords:** Embeddings, Machine learning, Evolutionary information, Protein language models, Multiple sequence alignments, Protein structure prediction, Secondary structure, Machine learning, Bioinformatics, Structural biology, Protein sequence analyses

## Abstract

Embeddings from protein Language Models (pLMs) are replacing evolutionary information from multiple sequence alignments (MSAs) as the most successful input for protein prediction. Is this because embeddings capture evolutionary information? We tested various approaches to explicitly incorporate evolutionary information into embeddings on various protein prediction tasks. While older pLMs (SeqVec, ProtBert) significantly improved through MSAs, the more recent pLM ProtT5 did not benefit. For most tasks, pLM-based outperformed MSA-based methods, and the combination of both even decreased performance for some (intrinsic disorder). We highlight the effectiveness of pLM-based methods and find limited benefits from integrating MSAs.

## Introduction

### MSA + ML = better protein prediction

Three decades ago, the combination of machine learning^1^ and evolutionary information derived from multiple sequence alignments (MSAs) brought about a breakthrough in protein secondary structure prediction^2,3^, i.e., of 1D-structure^4^. Predictions improved even more for larger families, increasing evolutionary information through diversity^5,6^. The idea was applied to other aspects of structure prediction in 1D^7–10^ and 2D^11^; it was refined for 2D predictions through evolutionary couplings^12–14^ and, most recently, with a leap in 3D structure prediction through DeepMind’s AlphaFold2^15^. The coupling of MSAs and ML was at the root of almost all state-of-the-art (SOTA) protein prediction methods published since the introduction of this concept 30 years ago^2,3^.

### Embeddings from protein language models (pLMs) replace traditional input

Over the last four years, novel representations, dubbed embeddings, for proteins have emerged from converting models developed for natural language processing (NLP) into protein language models (pLMs)^16–22^. Embeddings are learned solely from raw protein sequences without requiring additional phenotype annotations (self-supervised) using either auto-regressive pre-training or masked language modeling. Pre-learned embeddings from so called foundation models have successfully been input to a second level of protein prediction tasks (supervised training), surpassing MSA-based SOTA methods^17,23–28^. For some tasks, embeddings are so informative that even extremely shallow models (few free parameters), such as logistic or linear regression, suffice for the second-step supervised training^25,26^. In fact, raw embeddings capture information about inter-residue distance constraints without ever having encountered any structural information during training^28^. Besides improved performance and ease of adaptation to various tasks (ease of use), embedding-based methods often also speed up predictions. In computational biology, most resources are needed for inference, i.e., for applying methods to new proteins with Internet prediction servers such as *PredictProtein*^29^ completing over 10^6^ (10 M) predictions over its 32 years^2,29^. Thus, the pLM pre-training costs that must be done only once becomes less crucial. If embedding-based methods outperform MSA-based methods, does this imply that pLMs capture evolutionary information? Or do pLMs and MSAs correlate because they capture similar constraints on the observed protein sequences? If embeddings did not capture evolutionary information, we could improve predictions by combining embeddings with evolutionary information.

Here, we hypothesized that embedding-based methods could be improved through evolutionary information. We trained five similar architectures on different amino acid sequence representations and predicted protein secondary structures to test our idea. To forfeit bias from particular pLMs, we tried our methodology on three embedding types: SeqVec^16^, ProtBert-BFD^17^ (ProtBert), and ProtT5-XL-U50^17^ (ProtT5). We extended our analysis by comparing embedding- and MSA-based methods for various representative prediction tasks.

## Results

### MSAs can improve embedding-based predictions

**Fig. 1:**
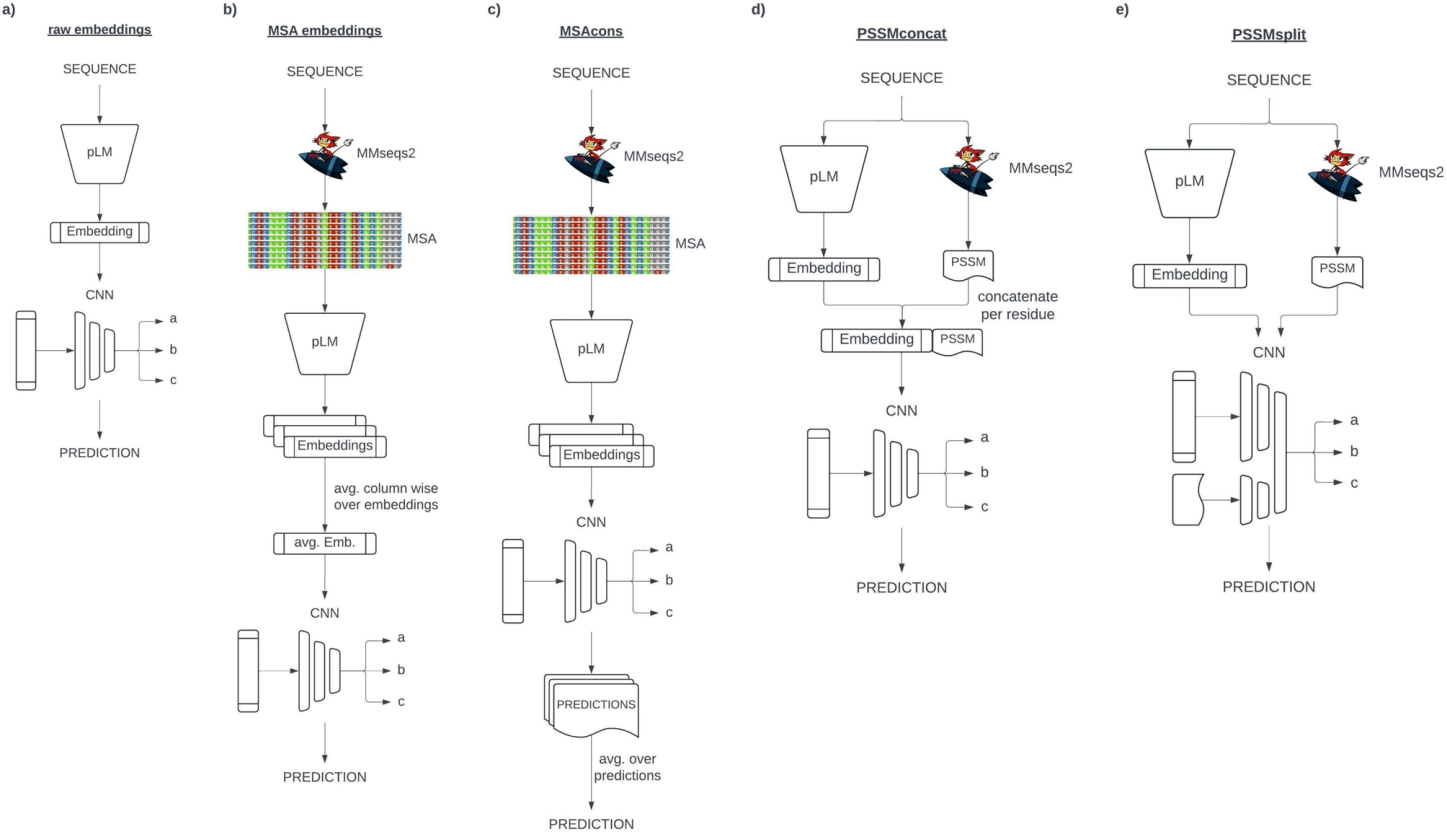
Prediction workflows. Schematic overview of the five main approaches for comparing the addition of evolutionary information to an embedding baseline. (**a**) *Raw embeddings* The query sequence is encoded with a pLM to obtain an embedding representation. This embedding is fed into a CNN to generate task-specific predictions. (**b**) *MSA embeddings* For each query sequence, an MSA is generated using MMseqs2^30^. Each sequence in the MSA is embedded with a pLM and the embeddings are averaged column wise according to the MSA alignment. This averaged embedding is fed into a CNN to generate task-specific predictions. (**c**) *MSA Consensus (MSACons)* An MSA is generated for each query sequence using MMseqs2^30^. Each sequence in the MSA is embedded with a pLM and each sequence embedding is feed into a CNN to make predictions for each sequence in the alignment. Final predictions for the query sequence are obtained by averaging predictions column-wise, based on the MSA. (**d**) *PSSMConcat* The query sequence is encoded with a pLM to obtain an embedding representation. Additionally a MSA is generated using MMseqs2^30^, from which a PSSM is computed. The per-residue embedding and PSSM features are concatenated into feature vectors, which are fed into a CNN to generate task-specific predictions. (**e**) *PSSMSplit* The query sequence is encoded with a pLM to obtain an embedding representation. An MSA is also generated using MMseqs2^30^, from which a PSSM is computed. The embedding and PSSM are provided as separate input features to a single CNN to generate task-specific predictions.

We generated two types of embeddings for each of the three investigated pLMs (SeqVec^16^, ProtBert^17^, and ProtT5^17^): *MSA embeddings* and *raw embeddings*. We refer to the embeddings explicitly enriched by evolutionary information derived from multiple sequence alignments (MSAs) as to *MSA embeddings* as opposed to the unaltered *raw embeddings*. We explicitly added evolutionary information in different ways. Firstly, by adding the readout from MSAs, namely the position-specific scoring matrix (PSSM), as an additional input feature for the supervised training (dubbed *PSSMSplit* and *PSSMConcat*). The main difference between the two PSSM-based approaches is in the way the prediction models use the input features: While PSSMConcat concatenates the raw query sequence embedding with the PSSM features and uses them together as the single model input vector, PSSM split uses raw embeddings and PSSM features as separate input features, only concatenating learned features from the different inputs at a later layer (Figs. S7, S8). Secondly, we added evolutionary information by generating MSAs for our query sequences and making predictions for each sequence in the alignment. To obtain the prediction for our query sequence, we averaged over predictions for all proteins in the MSA (dubbed *MSACons*). Thirdly, we generated embeddings for all sequences in the MSA of a query protein and averaged over the embedding vectors by columns in the MSA. This averaged embedding was then used as input for subsequent methods (MSA embeddings). Figure 1 provides a schematic overview of the employed approaches. We first studied the effect of these alternatives using the well-understood problem of secondary structure prediction in three states (H: helix, E: strand, and O: other (**¬**H **∧** ¬E).

**Fig. 2:**
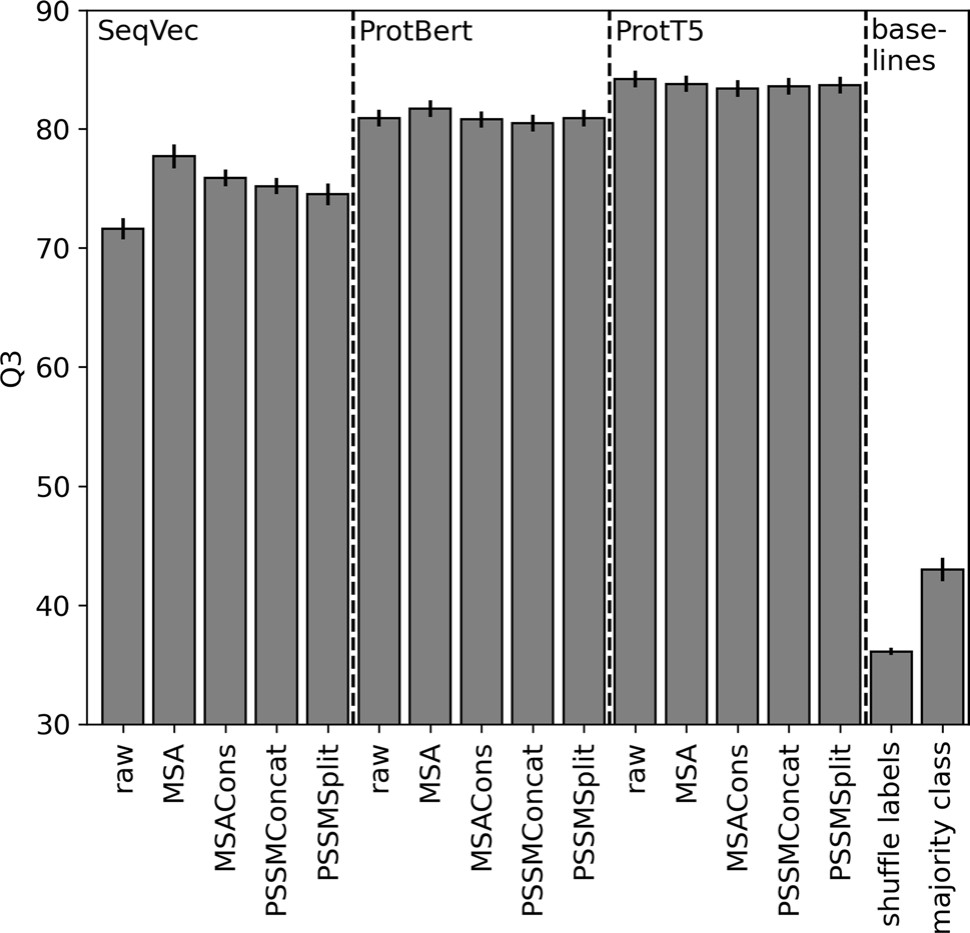
Secondary structure prediction. We tested transfer learning to predict protein secondary structure (in three states: helix, strand, other) with three pLM “generations”: SeqVec^16^, ProtBert^17^, ProtT5^17^. For each, we showed five alternatives: *raw* the original raw embeddings, *MSA* embedding values averaged over all proteins (in family as defined by the MSA), *MSACons* average over pLM-based prediction for each protein, *PSSMConcat* raw embeddings concatenated by PSSM, *PSSMSplit* raw embeddings and PSSM processed by disjunct model layers. *Baseline performance* approximated random predictions by: *shuffle labels* random draws from class distribution, and *majority class* predicting all residues in class *other*. The three-state per-residue accuracy (Q3) provided for the TEST100 data set. Error bars mark 1.96% standard errors, i.e., the 95% confidence interval (CI).

For SeqVec^16^ and ProtBert^17^ embeddings, the explicit addition of evolutionary information improved secondary structure prediction significantly (at 95% confidence interval—CI ± 1.96 stderr) of up to six percentage points for the simpler SeqVec and almost one for the more advanced ProtBert (assessed by the three-state per-residue accuracy Q3 Fig. 2, Table S1). In contrast, MSAs did not improve for ProtT5 (Fig. 2, Table S1). PSSM-based models and averaging predictions for all proteins in an MSA (MSACons) remained numerically even below the raw embeddings (Fig. 2, Table S1; the decrease was not statistically significant). Visualizing the CNN layers that combine PSSM-based and embedding-based features in our PSSM-based models (PSSMConcat, PSSMSplit) revealed that weights applied to PSSM features are often strongly negative or positive for all embedding types, indicating that the models actively use these features (Figs. S2, S3), even if no significant performance changes could be observed.

**Fig. 3:**
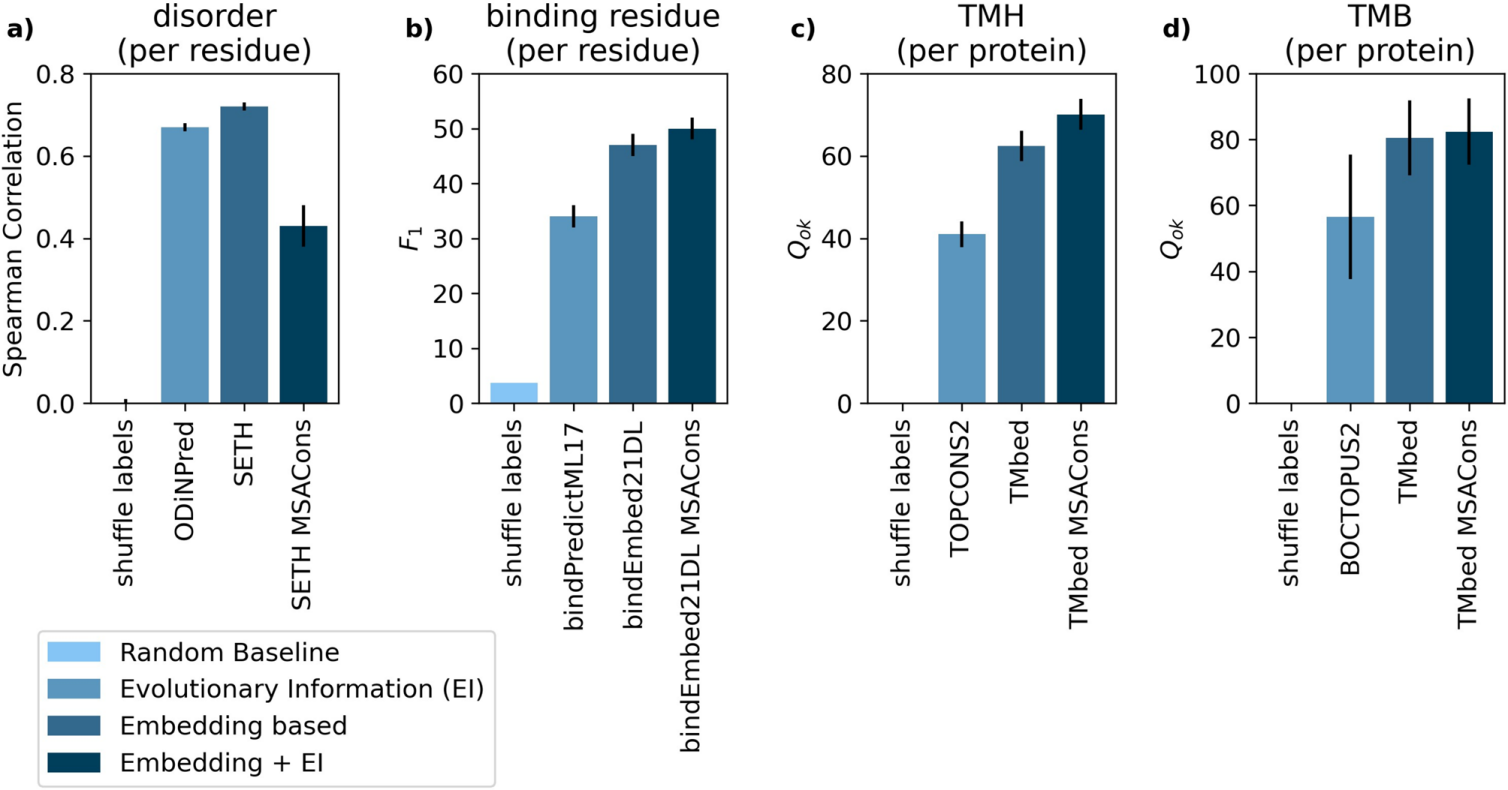
Raw embeddings vs MSACons. Explicitly adding embeddings through multiple sequence alignments (MSAs, here MSACons) slightly increased performance for three of the four representative prediction tasks (binding, TMH and TMB). Error bars mark 1.96% standard errors, i.e., the 95% confidence interval (CI). (**a**) Per-residue Spearman correlation for disorder prediction on the CheZOD117^43^ dataset; (**b**) per-residue binding residue F1 prediction performance on the TestSet225^24^; (**c**) per-segment Q_ok_ for TMHs on 571 helical transmembrane proteins^23^; (**d**) per-segment Q_ok_ for TMB on 57 beta-barrel membrane proteins^23^. The original MSA-based methods were (left to right): ODINPred^43^, bindPredictML17^34^, TOPCONS2^35^, BOCTOPUS^36^; the original embedding-based methods: SETH^26^, bindEmbed21DL^24^, TMbed^23^.

The prediction of intrinsically disordered regions in proteins^31^ is a task for which MSA-based predictions are among the top performing methods despite the difficulties of aligning such proteins^32,33^. Nevertheless, embedding-based methods such as SETH, that purely rely on single sequence input, perform similarly^26^. For this task, we compared to the MSA-based ODiNPred. The MSA-augmented SETH MSACons performed significantly worse than the original SETH^26^ (Fig. 3a).

In the past, our group has found that predicting which residues in a protein bind to non-protein substrates is particularly challenging due to the scarcity of reliable experimental data^34^. For this task, we compared the embedding-based bindEmbed21DL^24^, and the MSA-based bindPredictML17^34^ to adding MSA information. Here, augmenting bindEmbed21DL with evolutionary information (MSACons) improved numerically over the original bindEmbed21DL (bindEmbed21DL^24^ vs. bindEmbed21DL MSACons, Fig. 3b). However, this difference was not statistically significant.

Our final prediction task, in comparing inputting raw embeddings with combining embeddings with evolutionary information, was the prediction of transmembrane segments. We compared top MSA-based methods such as TOPCONS2^35^ (for transmembrane helices, TMHs) and BOCTOPUS2^36^ (for transmembrane beta-strands, TMB) with the embedding-based TMbed^23^. All of those successfully predict transmembrane segments^37–39^. We found that the per-segment Q_ok_ (“Methods” section) slightly improved through MSACons over the original TMbed. For TMHs, this improvement was statistically significant (TMbed^23^ vs. TMbed MSACons, Fig. 3c,d).

Additionally, to the discussed tasks we also evaluated our approach on predicting the conservation of residues within a protein family based on the embedding based VESPA^25^ approach. Due to issues discovered with the reproducibility of VESPA’s reported performance, details for this task are provided in the SOM (SOM Sect. 1.2 Conservation Prediction).

For all four tasks collected to capture varying aspects of protein prediction, embedding-based methods outperformed or were on par with the best MSA-based methods. Averaging over predictions for all proteins in an MSA (MSACons) improved statistically significantly for TMH predictions; it improved numerically for predicting TMBs and binding residues. In contrast, averages were significantly lower for disorder prediction (at 95% confidence interval—CI ± 1.96 stderr).

### Raw embeddings mostly outperformed evolutionary information

**Fig. 4:**
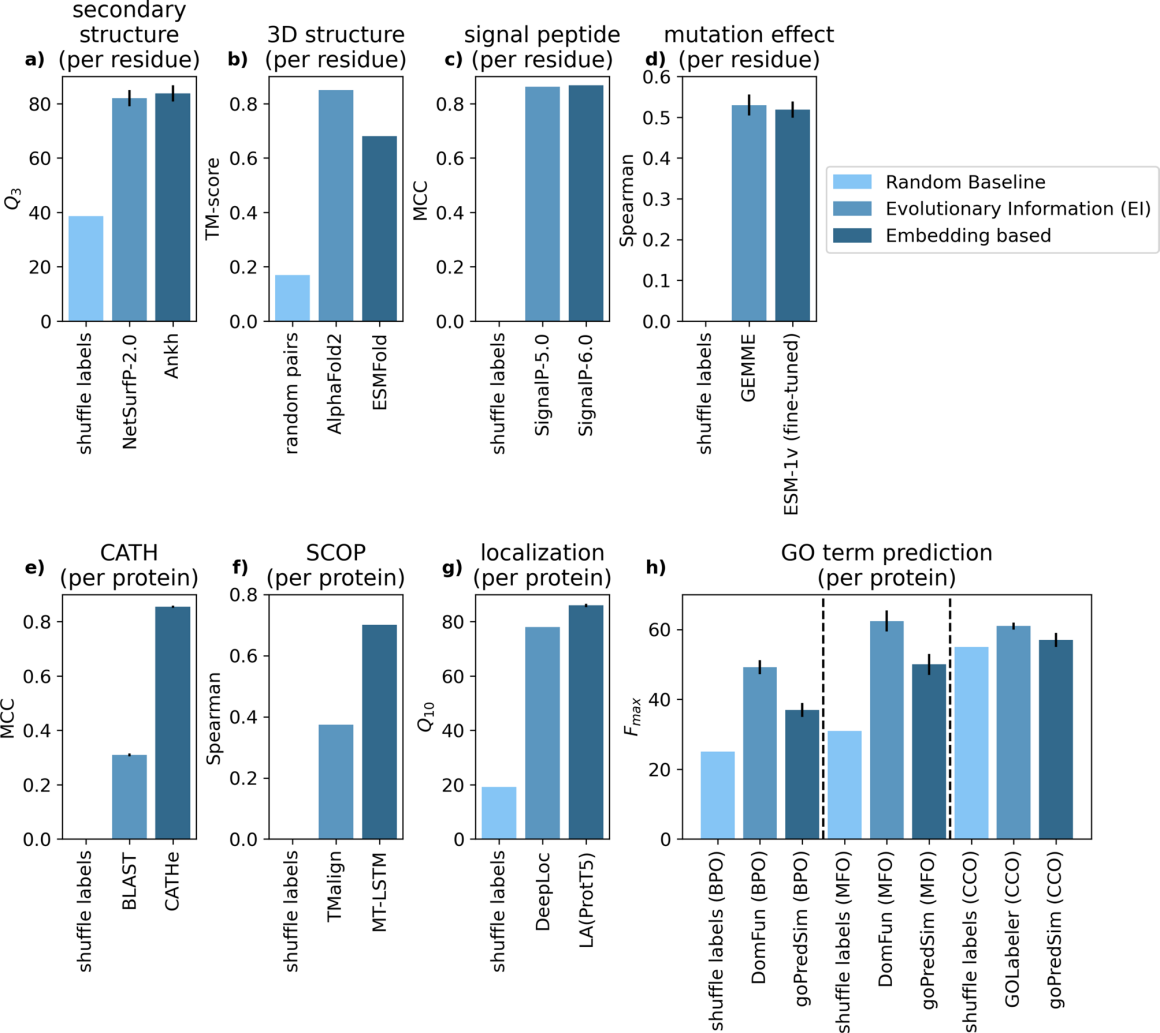
Literature MSA vs. embedding. We compared the performance of MSA-based to embedding-based methods published for seven different protein prediction tasks (baselines: *shuffle labels*: random draws from class distribution and *random pairs*: average TM-score of randomly picking two unrelated proteins). In each panel, values referred to the same dataset and only the numerically best performing method shown (more methods in Tables S9–S13). Error bars mark 1.96% standard errors, i.e., the 95% confidence interval (CI), if available in the original literature. The tasks and methods were: (**a**) per-residue Q_3_ performance for secondary structure prediction on CASP12 dataset^54^ (methods: NetSurfP-2.0^55^, Ankh^56^). (**b**) Per-residue TM-score average for 3D structure prediction on the CASP14 dataset^57^ (methods: AlphaFold2^15^, ESMFold^58^). (**c**) per-residue MCC performance for signal peptide prediction on SignalP-5.0 benchmark^59^ (methods: SignalP-5.0^59^, SignalP-6.0^40^). (**d**) Per-residue Spearman correlation of mutation effect prediction on the DMS41^60^ (methods: GEMME^61^, ESM-1v (fine tuned)^62^). (**e**) Per-protein MCC performance for CATH prediction on the TOP1773^41^ (methods: BLAST^63^, CATHe^41^). (**f**) Per-protein Spearman correlation for SCOP class prediction on ASTRAL 2.06^42,64^ (methods: TMalign^65^, MT-LSTM^42^). (**g**) Per-protein Q_10_ performance on localization prediction on the DeepLoc test set^66^ (methods: DeepLoc^66^, LA(ProtT5)^27^). (**h**) per-protein F_max_ score for GO term prediction on the CAFA3 dataset^50^ (BPO: biological process ontology; MFO: molecular function ontology; CCO: cellular component) (methods: DomFun^51^, goPredSim^52^, GOLabeler^53^).

Comparing values recently published, pLM-based methods outperform or match MSA-based solutions for many protein prediction tasks. On the per-residue level, these include the prediction of secondary structure (Fig. 4a, Table S9), signal peptides^40^ (Fig. 4c, Table S11), binding residues (Fig. 3b, Table S7), and disordered regions (Fig. 3a, Table S6). On the per-protein level, these include the prediction of CATH-classes^41^ (Fig. 4e, Table S13), SCOP classes^42^ (Fig. 4f, Table S14), and subcellular location^27^ (Fig. 4g, Table S15). On the per-segment level, these tasks include transmembrane prediction (TMHs: Fig. 3c, Table S8; TMBs: Fig. 3d, Table S8).

The opposite (evolutionary information more accurate than embeddings) remains true for 3D structure prediction^44^ (Fig. 4b, Table S10), possibly because AlphaFold2^15^ has optimized so many aspects of this task in ways not readily transferrable to using pLMs as input^45^. Similarly, the best evolutionary information-based methods still appear to outperform pLM-based methods in predicting the effect of point mutants (SAV: single amino acid changes; Fig. 4d, Table S12) as measured by Deep Mutational Scanning (DMS/MAVE) experiments^25,46–48^ and for predicting aspects of function as categorized by the GeneOntology (GO) numbers^49^ (Fig. 4h, Table S16) For both of these problems, advanced machine learning models on top of MSAs continue to outperform simple pLM-based solutions, for the time being^50–53^. However, for both problems, embedding-based methods, such as VESPA^25,46^ and goPredSim^52^, come close without using MSAs.

## Discussion

We hypothesized that explicitly using evolutionary information from multiple sequence alignments (MSAs) when inputting embeddings from large protein Language Models (pLMs) could improve the performance of subsequent supervised learning protein prediction tasks. While we proved this hypothesis for earlier pLMs, such as SeqVec^16^ and ProtBert^17^, we failed to improve for embeddings from more advanced pLMs, such as ProtT5^17^ (Figs. 2, 3, 4, Tables S5–S16). The likely interpretation of the first finding (improvement through explicitly using MSAs) is that those first pLM-generations either failed to capture evolutionary information or did not capture it to the same degree as MSAs. The gradual increase in the power of transfer learning (using the “lessons learned” by pLMs, i.e., the *embeddings*, as input to subsequent supervised training) with each new pLM generation suggested that pLM embeddings increasingly capture information correlated with evolutionary information taken from MSAs.

### Do embeddings capture or correlate with evolutionary information?

The difficult question then becomes: do embeddings from advanced pLMs correlate with evolutionary information, or do they explicitly capture it? Understanding this distinction is important because it impacts how we interpret the utility of embeddings and the underlying insights they provide.

Biologically, correlation would imply that embeddings reflect biophysical and functional constraints on protein sequences. Examples of those include inter-residue bonds, the balance between hydrophobic and hydrophilic residues^67^, and binding sites. These constraints are reflected in both MSAs and embeddings from advanced pLMs. This suggests that pLMs can learn the underlying grammar of protein sequences, which also constrain MSAs. We conclude that embeddings and MSAs may be correlated because they both reflect these constraints.

Our results did not decisively support or invalidate either hypothesis. We considered several approaches to explore this further. Firstly, we could evaluate if differences in family size result in prediction differences. Previous studies have observed such differences^17,46,68^. However, the scarcity of high-resolution data for small families and biophysical differences between small and large families further complicate this analysis. For a more in-depth discussion of this, see SOM Sect. 2.1. Limitations of family size.

Secondly, we could investigate the impact of training protein language models without evolutionary information. A pLM trained on large databases like BFD^69^ may implicitly learn the same constraints captured in an MSA simply by encountering sequences from larger families more frequently. However, training pLMs without this evolutionary information is currently impractical due to data limitations. We provided further details on this in SOM Sect. 2.2. Limitations of Training pLMs without Evolutionary Information. Reducing the data used during pLM training to only non-redundant sequences would require much larger databases than are currently available. This issue makes this approach unfeasible in the near future.

Thirdly, systematically biasing training of pLMs towards a specific family could help answer the question if pLMs capture or correlate with the evolutionary information contained in MSAs. However, this approach exceeds our computational resources and may introduce confounding factors that would make drawing conclusions difficult. For more details, see SOM Sect. 2.3. Limitations of biasing pLM training towards Families.

Given these challenges, the most practical way of answering our question was by evaluating if explicitly adding evolutionary information in the form of MSAs or PSSMs improves embedding-based predictions. Our results imply that embeddings capture information that at least correlates with the information in MSAs. Adding MSAs to the embedding-based approaches did not consistently enhance performance. Ultimately, further research on whether embeddings capture or correlate with evolutionary information can help guide future research and tool development. If embedding-based methods only correlate, improving MSA-based methods could still be valuable. If embeddings capture evolutionary information, this may shift the focus in our field toward further development and optimization of pLMs.

### Impact of evolutionary distance on prediction performance

To what extent evolutionary information aids in predicting specific protein properties depends on the distance over which these properties are conserved as well as the sequence similarity in the MSA or PSSM, relative to the query sequence. For example, binding residues are not expected to be conserved well below 75% sequence identity^70^ and will benefit minimally from highly diverse alignments but may benefit from highly similar aligned sequences. Conversely, protein structure is still conserved at thresholds as low as 30% sequence similarity^70,71^ and is therefore expected to benefit more from diverse MSAs. Similarly, the diversity of evolutionary information captured by or correlated with embeddings is likely to impact which tasks are best suited for embedding-based models. However, the diversity of evolutionary information encoded in embeddings is currently hard to ascertain. These differences are critical for understanding the varied impact of evolutionary information and embeddings across different prediction tasks and should be considered in future model development and evaluations.

### Performance gains for embedding-based methods only for early-generation pLMs

For secondary structure prediction, the advance from relatively simple pLMs such as SeqVec^16^ (based on ELmO^72^ predicting the next word/amino acid in a sentence/protein sequence) to more advanced ProtBert^17^ (based on BERT^73^) sufficed to turn the gain from explicitly using MSAs into becoming a numerical improvement that remained statistically insignificant (Fig. 2). Stepping from BERT to T5^74^ (or more precisely, ProtBert to ProtT5^17^), the numerical advantage (ProtBert, Fig. 2) turned into a numerical disadvantage (ProtT5, Fig. 2) of using MSAs. One of the major aspects in the advance of T5 and BERT over, e.g., ELMo, is the concept of attention heads^75^. This explained why the accurate prediction of protein 3D structure from sequence leaped in the transition from SeqVec to ProtBert and ProtT5, as shown recently^28^. However, we did not use these attention heads for secondary structure prediction. Thus, the rise in secondary structure prediction performance to a similar level with and without explicitly using MSAs (Fig. 2, Tables S1, S2) implied that especially ProtT5 additionally extracted other constraints from protein sequences relevant for structure formation.

### MSA average worsens the prediction of disorder

When evaluating the performance of integrating evolutionary information into existing embedding-based methods by averaging over all single-protein predictions in an MSA (MSACons), we observed that only one of the methods showed a significant improvement (Fig. 3, Tables S5–S16). For the per-residue prediction of disorder (as measured by CheZOD scores^76^), the MSA-average over predictions (MSACons) significantly decreased performance over the original method (Fig. 3a, Table S6).

This decrease may be related to the difficulty of correctly aligning intrinsically disordered proteins (IDPs)^77–79^. Disordered regions often have lower conservation and evolve faster, leading to higher variability and potential misalignments^80,81^. Incorrectly aligned proteins introduce noise, which can degrade the performance of methods relying on MSA-derived features. Furthermore, with the evolutionary conservation of disordered regions being less stringent, orthologs are frequently less similar^80,81^, challenging the conceptual foundation that MSA information would consistently improve disordered regions. While there are tools specialized in accounting for these challenges, we adhered to our MMseqs2^30^ workflow to ensure consistency across all prediction tasks. Adjusting our approach to optimize MSA generation specifically for disordered proteins would require extensive research, potentially warranting a separate publication.

### Similar or better performance for embedding-based methods on most prediction tasks

Our method comparison of embedding-based and evolutionary information-based methods in the literature shows that embedding-based models are as accurate or better performing for most tasks (Figs. 3, 4, Tables S5–S16). The only three exceptions in our set constituted tasks related to predicting protein function (Fig. 4h, Table S16), the effect of sequence variation upon molecular function (Fig. 4d, Table S12) and 3D structure prediction (Fig. 4b, Table S10).

Most proteins consist of several structural domains^82–84^. Recent state-of-the-art methods predict aspects of function based on domain information by first chopping full-length proteins into their domain constituents and then learning patterns in these units, e.g., DomFun^51^ or the recent breakthrough method ChainSaw^85^. In contrast, the embedding-based goPredSim transfers annotations from a protein of known function to a query protein through the similarities of per-protein embeddings; in a method referred to as embedding-based annotation transfer (EAT)^52^. The domain-chopping of DomFun might ultimately be more relevant for its edge over goPredSim (Fig. 4h, Table S16), basing its decision on full-length proteins rather than the aspect of explicitly using evolutionary information. In fact, embedding-based protein comparisons substantially drop in performance when using full-length proteins rather than domains^86^.

Methods predicting the effect of sequence variation upon protein function have recently been assessed through the lens of deep mutational scanning (DMS)^25,43,46^. Most of those analyses suggested that no prediction method consistently outperforms all others^25,46,61^. While DMS data continue to improve, resources are still limited due to the enormous experimental complexity. Alternative data sets come with their own issues, e.g., most existing methods have been over-trained to fit small data sets, yet other data sets heavily depend on creating MSAs. Given all these issues, whether MSA-based methods outperform pLM-based solutions in predicting the effect of sequence variation upon molecular function remains to be seen.

This left no example other than 3D structure prediction (Fig. 4b, Table S10), for which MSA-based methods (i.e. AlphaFold2^15^), outperformed pLM-based methods. Once again, it is too early to tell whether or not this roots in the fact that the developers of this unique breakthrough engineered so many outstanding solutions for so many aspects of the structure prediction challenge. Overall, pLMs already extract important information from MSAs. With every generation of pLMs advancing significantly^45^, the point at which pLMs can essentially replace MSAs as input for all aspects of protein prediction appears to be getting closer. Clearly, pLMs have become too advanced to benefit from simple MSA-averaging (Fig. 2, Tables S1, S2). However, the lack of improvement does not add any evidence to answer whether embeddings capture evolutionary information directly or whether they, instead, capture the constraints underlying the existing sequences as much as MSAs. Ultimately, this distinction may not matter in practice as long as pLMs effectively replace MSAs for protein prediction.

## Conclusions

Over the past three decades, the evolutionary information captured in multiple sequence alignments (MSAs) of protein families has become the most successful tool in combination with machine learning to predict aspects of protein structure and function. Recently, embeddings from protein Language Models (pLMs) have replaced MSA as standard input for protein prediction. For many tasks, they reach or beat MSA-based predictions. What if we combined MSAs and embeddings? We tested various approaches to incorporate evolutionary information into embeddings. While for earlier pLMs such as SeqVec^16^ and ProtBert^17^ the combination significantly improved accuracy, the more recent pLM ProtT5^17^ did not benefit from adding MSAs (Figs. 2, 3, 4). Is this because pLMs capture evolutionary information, or because prediction methods reached some level of saturation? Embedding-based often outperform MSA-based methods (Fig. 4, Tables S5–S16). Naturally, better-performing methods are closer to the theoretical performance limit or saturation points than those that perform worse. Except for this obvious observation, we have no evidence to claim that saturation explained our findings. Combining MSAs and embeddings even decreased accuracy for disorder, possibly due to bias introduce in the way we created MSAs. However, we could not find any evidence to solve the open question whether pLMs capture evolutionary information contained in MSAs directly or whether pLMS simply correlate with evolutionary information by capturing the same underlying constraints on sequence space (all observed sequences) in a similar way as MSAs do. Overall, our study highlights the effectiveness of embedding-based methods.

## Methods

### Data set—secondary structure prediction

For 156,897 proteins (≤ 3.5 Å, ≥ 40 residues), we extracted sequences from the PDB^87^. At the time of data generation (2020-10-26), this yielded 573,479 chains that cross-checked with PDBredo DB^88^ and CATH^89^ by computationally removing any IDs unavailable in either of those databases. This kept 296,596 protein chain sequences from 117,623 different proteins.

All remaining sequences were pairwise aligned by MMseqs2^30^. For each pair, we computed the HSSP-value (HVAL)^71,90^ (Eq. 2) and the percentage pairwise sequence identity (PIDE, Eq. 1) as follows^91^:

1 $$PIDE=\frac{n\_ident\times 100}{L},$$

2 $$HVAL=PIDE-\left\{\begin{array}{ll}100 & \quad for\, L \le 11 \\ 480\times {L}^{-0.32 \times \left[1+\text{exp}\left(-L\div 1000\right)\right]}& \quad for\, 11<L \le 450 \\ 19.5 & \quad for\, L >450 \end{array}\right.,$$

where L is the number of M-states, and n_ident is the number of identical residues in the alignment. Note that the HVAL computes proximity for protein pairs based only on their sequences.

### Training, validation, and test set (secondary structure prediction)

Given that our prediction task is structure-based, we used the HVAL to reduce the redundancy between the separate data sets used for training, validation, and final performance assessment (test) to account for and remove structural similarity: TEST100 was used only to assess performance, and VAL100 was used for optimizing hyper-parameters. We chose these sets as follows:

- *TEST100* we randomly selected 100 sequences meeting three criteria: (1) Deposited after April 2018 to allow a fair comparison to other recent methods, (2) resolution ≤ 2 Å, (3) any sequence pair (a,b) with a,b ∈ TEST100 must have an HVAL ≤ 0 (Eq. 2).
- *VAL100* we randomly selected an additional 100 sequences constrained to (1) deposited before April 2018, (2) resolution ≤ 2 Å, and (3) we ascertained that any sequence pair (a,b) with either a ∈ TEST100 or a ∈ VAL100 and b ∈ VAL100 had a maximal HVAL ≤ 0.
- *TRAIN6727* we used the remaining sequences for training if and only if the following criteria were fulfilled: (1) deposition before April 2018, (2) CATH annotations on the topology level (T) had to be different from any contained in TEST100 or VAL100, (3) HVAL ≤ 0 for any pair (a,b) with a ∈ TEST100 or a ∈ VAL100 and b ∈ TRAIN6727, and (4) PIDE ≤ 70 as computed by MMseqs^30^ for any pair (a,b) with a,b ∈ TRAIN6727, if a ≠ b. This yielded 6727 protein chains for training.

### Preventing data leakage and ensuring robustness

To prevent data leakage between training, validation and test set, we used an HVAL ≤ 0. This cutoff roughly corresponds to a maximum sequence similarity of 30% or lower but additionally ensures that no pair of sequences share similar structures^71^. While this approach minimizes the risk of overlap, we acknowledge that sequences in MSAs could exhibit higher pairwise similarities and have a higher HVAL. To ensure that no data leakage was introduced in this way, we performed pairwise global alignments for training, validation and test sequences, including the ones introduced by the MSAs. This included aligning all training sequences to all validation sequences, all training sequences to all test sequences and all validation sequences to all test sequences. Our analysis of the resulting sequence similarities confirmed that even with the sequences introduced through MSAs, all pairs between these sets fell below the threshold of 0.3. Furthermore, our analysis with ProtT5^17^ and ProtBert^17^ embeddings show no significant performance gains for MSA or PSSM models (Fig. 2, Table S1). If there were data leakage, noticeably increased performance would be expected for any evolutionary information-based model. The lack of such a change confirms that no data leakage was introduced. To ensure the robustness and generalization capability of our models we evaluated our top-performing model (ProtT5 raw embeddings) on the CASP12 dataset^54^. Our model achieved performances consistent with previous methods (Table S9), further validating the robustness of our approach and the effectiveness of our data splits.

### Secondary structure states

We reduced the eight classes of DSSP^92^ secondary structure assignments to three states through the following standard protocol^3^: Alpha helix classes DSSP-H, DSSP-G, and DSSP-I to helix (H), beta-strand classes DSSP-E and DSSP-B to strand (E), and all remaining classes to other (“–” or “ ”, often misleadingly referred to as *loop*).

### Data sets—MSA-based averages

We obtained the datasets originally used to estimate performance to appropriately compare adaptations with original methods. To provide a comprehensive overview of our approach, we have summarized the datasets and methods used for each prediction task in Table 1. For VESPA^25^, we used the ConSurf10k^25^ test set (https://zenodo.org/record/5238537) with per-residue conservation scores in 9 classes (1: most variable to 9: most conserved) for 519 sequence unique proteins. For SETH^26^, we used the CheZOD117^43^ set introduced along with ODiNPred^43^ with per-residue CheZOD scores for 117 proteins. The CheZOD score quantifies the extent of residue disorder by comparing the nuclear magnetic resonance spectroscopy-derived chemical shift values^93^ with the computed random coil chemical shifts^94^. For bindEmbed21DL^24^, we used TestSet225^24^ (https://github.com/Rostlab/bindPredict) with per-residue annotations for 225 proteins (classified as binding metal, nuclear or small molecules, or are non-binding). For TMbed^23^, we used 571 transmembrane proteins (TMPs) with transmembrane alpha helices (alpha-TMP)^23^ and the 57 TMPs with transmembrane beta strands (beta-TMP)^23^ (https://github.com/BernhoferM/TMbed) with per-residue annotations for four states: TM alpha-helix, TM beta-strand, signal peptide, none, as well as, of so-called membrane topology (inside/outside).

**Table 1:** Overview of prediction tasks, dataset and approaches used for integrating evolutionary information.

Prediction task	Dataset	Approach for integration of evolutionary information
α-helix bundle transmembrane segments	571 α-TMPs^23^	MSACons
β-barrel transmembrane segments	57 β-TMP^23^	MSACons
Binding residue prediction	TestSet225^24^	MSACons
Conservation	ConSurf10k test^25^	MSAcons
Disorder	CheZOD117^43^	MSACons
Secondary structure prediction	TRAIN 6727, VAL100, TEST100	MSACons, PSSMSplit, PSSMConcat, MSA embeddings

Overview of the datasets used for testing the effect of integration of evolutionary information. The first column specifies the prediction task that was investigated, the second one the dataset used for evaluating the prediction performance and the last column what kind of approach(s) were used for integration of evolutionary information.

### Data sets—literature

We obtained additional datasets to compute random baselines and compare recent MSA- and pLM-based methods. In particular, we used for per-residue predictions: secondary structure: CASP12^54^ dataset (20 unique sequences, https://www.dropbox.com/s/te0vn0t7ocdkra7/CASP12_HHblits.csv?dl=1), 3D structure: CASP14^57^ dataset (51 unique proteins, https://predictioncenter.org/download_area/CASP14/), signal peptides: SignalP-5.0 benchmark^59^ set (8811 unique sequences, https://services.healthtech.dtu.dk/services/SignalP-5.0/benchmark_set.fasta), SAV effect prediction: DMS41^60^ dataset (41 unique proteins, https://static-content.springer.com/esm/art%3A10.1038%2Fs41592-018-0138-4/MediaObjects/41592_2018_138_MOESM4_ESM.xlsx). For per-protein predictions, we obtained: CATH superfamily: TOP 1773^41^ dataset (6712 unique sequences, https://zenodo.org/record/6327572), SCOP class: ASTRAL 2.06 test^19,64^ set (5602 unique sequences, http://bergerlab-downloads.csail.mit.edu/bepler-protein-sequence-embeddings-from-structure-iclr2019/scope.tar.gz), GO function: CAFA3^50^ dataset (130,827 unique proteins, https://biofunctionprediction.org/cafa-targets/CAFA3_targets.tgz), and for subcellular: location: DeepLoc Test^59^ set (2889 unique sequences, https://services.healthtech.dtu.dk/services/DeepLoc-1.0/deeploc_data.fasta). Although we provided some figures for the sizes of those data sets, those numbers are not strictly comparable. For one task, N proteins might capture all aspects of prediction methods; for another, M >> N might still not suffice. Similarly, head-on comparisons between the accuracy of tasks T1 and T2 are potentially very misleading. For guidance, we provided values reflecting similar solutions toward random baselines. (Even improvement over random is irrelevant as a means of comparison, as illustrated by the following example: assume task 1 (T1) having three equally distributed states (random Q3 = 33%), and task 2 (T2) to have ten equally distributed states (random Q10 = 10%). Imagine a method three times better than random for T1 (Q3 = 3 × 33 = 100%) and another reaching the same gain over random for T2 (Q10 = 3 × 10 = 30%).

T1 solved by a method reaching Q3 = 100% (3 times better than random). The method for T1 is completely right, while that for T2 might be completely blind on 4 of the ten states and still only get “half” of the predictions right.)

### MSA and PSSM

We created multiple sequence alignments (MSAs) by running MMseqs2^30^ against UniRef50^95^ for all query proteins. MMseqs2^30^ was run with a sensitivity (-s) of 7.5 for the prefiltering module, a minimum sequence identity (–min-seq-id) of 0.2, and 3 iterations (–num-iterations). MMseqs2 first selects only cluster representatives and then extends the results by adding the most diverse sequences to the alignment. Position-specific scoring matrices (PSSMs) were also obtained from MMseqs2. This workflow was consistently applied to all tasks that required MSA or PSSM generation, namely: secondary structure (TRAIN 6727, VAL100, TEST100), transmembrane prediction (57 β-TMP^23^, 571 α-TMPs^23^), binding residues (TestSet225^24^), conservation (ConSurf10k test^25^) and disorder (CheZOD117^43^). For conservation prediction, we additionally obtained the precomputed MSAs of our query sequences from ConSurfDB^96^ that have been generated with MAFFT^97^.

### Embeddings

We used three different protein language models (pLMs) to generate our embeddings: The transformer-based ProtT5^17^ and ProtBert^17^ and the bi-directional pLM SeqVec^16^. For each model, we compared the following two alternatives.

#### Raw embeddings

For ProtT5 and ProtBert, we used only the encoder to convert each protein sequence into an embedding matrix (dimensions 1024 × L; L number of residues), representing each residue by a 1024-dimensional vector. For SeqVec, we extracted the forward and backward pass of the first LSTM layer (also dimension 1024 × L). We used the pre-trained models without any task-specific fine-tuning throughout.

#### MSA embeddings

Besides the embeddings for each query sequence Q, we computed embeddings for each protein in the MSA of Q. We removed gaps and mapped rare amino acids to X before the encoding. For each residue position *i* in Q, we averaged over all per-residue embeddings from any protein aligned at *i*.

### Prediction methods

We compared identical model architectures for three different 1024-dimensional input vectors: *raw embeddings*, *MSA embeddings*, and by the MSACons average over the raw embeddings for all proteins. Additionally, we tested two highly similar architectures to incorporate the additional PSSM input. Figure 1 provides a schematic overview of the used approaches. By comparing as similar architectures as possible, we ensured that performance differences could be primarily attributed to different inputs instead of model complexity, capacity, or other unknown confounding factors.

#### Input

On top of raw—and MSA-embeddings (both 1024 input units), we evaluated the combination of raw embeddings and PSSMs (PSSMSplit and PSSMConcat). The mixture models (embeddings + PSSMs) used 1024 (embedding dimension) + 20 (PSSM) input units (dimensions for PSSM; SOM: Figs. S5–S7).

#### Models

Our models consisted of multiple consecutive convolutional layers, each using leaky *relu* as activation function^98^. The input dimensions for the embedding-only models (MSACons, raw, and MSA embeddings were 1024, 32, 16, and 8, and the output layer had three units for the three secondary structure states (H: helix, E: strand, –: other; Fig. S6). Note the MSACons model applied the original method to predict for each sequence aligned in the MSA and then averaged over the predictions, while MSA-embeddings input the averaged embeddings into the original method. To combine PSSMs and embeddings, we tested two solutions. As none of those improved over simpler approaches, we confined the results to the SOM (Figs. S7, S8).

### Performance measures—secondary structure

We used 3-state per-residue accuracy (Q_3_, Eq. 3) as our main performance measure for secondary structure prediction evaluation:

3 $${Q}_{3}= \frac{\#residues\, predicted\, correctly}{\#total \,residues}\times 100.$$

Standard deviations and errors for all measures were estimated on a per-chain base by counting the total and correctly predicted number of residues in a chain and calculating the listed performance measures for each chain. The resulting distribution over all chains was used to compute the mean and standard deviation (SD). Standard error was calculated by dividing SD by the square root of the sample size (Eq. 4).

4 $$Standard\, Error\, \left(SE\right)= \frac{SD}{\sqrt{n-1}}.$$

Additional performance measures for secondary structure prediction evaluation have been confined to the SOM (SOM 3.3 Additional Performance Measures).

### Performance measures—other tasks

We evaluated embeddings-based methods following the original method publications. For conservation prediction, we used MCC as described in the SOM (Eq. S1); TP_x_: correctly predicted as conserved (> 5); FP_x_: predicted conserved but annotated as not conserved (< 6); TN_x_: correctly predicted as not conserved; FN_x_: predicted not conserved and annotated as conserved). The Spearman correlation p (Eq. 5) assessed disorder predictions:

5 $$Spearman\, Correlation \left(p\right)= \frac{\sum_{i=1}^{m}[({u}_{i}-\frac{1}{m}\sum_{j=1}^{m}{u}_{j})\times ({v}_{i}-\frac{1}{m}\sum_{j=1}^{m}{v}_{j})]}{\sqrt{\sum_{i=1}^{m}{({u}_{i}-\frac{1}{m}\sum_{j=1}^{m}{u}_{j})}^{2}\times \sum_{i=1}^{m}{({v}_{i}-\frac{1}{m}\sum_{j=1}^{m}{v}_{j})}^{2}}}.$$

The F1 score (Eq. 6) evaluated binding residue predictions:

6 $${F}_{1} = 2\frac{Recall\times Precision}{Recall+Precision},$$

7 $$Recall = \frac{TP}{TP+FN}\times 100\%,$$

8 $$Precision = \frac{TP}{TP+FP}\times 100\%.$$

With TP/FP: correctly predicted as binding/non-binding and FP/FN: incorrectly predicted as binding/non-binding.

Transmembrane predictions were compared based on a per-protein level. For this purpose, a segment was considered to be predicted correctly if (1) the start and end did not differ by more than five residues from the annotated segment and (2) the intersection overlap of the predicted and observed segment was at least half of their union^23^. We evaluated transmembrane prediction performance using Q_ok_ as described in Eq. (9):

9 $${Q}_{\text{ok}}= \frac{\# proteins \,with\, all\, segments \,of \,type \,T \,correct}{\# proteins\, with \,segments \,of \,type \,T}\times 100\%.$$

Q_10_ (Eq. 10) evaluated location prediction:

10 $${Q}_{10}= \frac{\# \,proteins\, predicted \,correctly}{\#\, total \,proteins}\times 100\%.$$

## Supplementary Information

Supplementary Information.

## Data Availability

The source code is publicly available as a GitHub repository at https://github.com/erckert/EV-embeddings. The dataset used for the comparison of different approaches to integrating evolutionary information for secondary structure prediction is publicly available at: 10.5281/zenodo.10026192.

## Abbreviations

CATH: Protein structure classification database
DMS: Deep mutational scanning
FN: False negatives
FP: False positives
GO: GeneOntology
HVAL: Distance to HSSP curve
HSSP curve: A curve that represents an empirically determined threshold for automatic family assignment
MCC: Matthews correlation coefficient
ML: Machine learning
MMseqs2: Many-against-many sequence searching tool
MSA: Multiple sequence alignment
NLP: Natural language processing
PDB: Protein Data Bank
PDBredo DB: A database for optimized crystallographic structure models
pLM: Protein language model
ProtBert: ProtBert-BFD protein language model
ProtT5: ProtT5-XL-U50 protein language model
PSSM: Position specific scoring matrix
Q3: 3-State accuracy
SD: Standard deviation
SE: Standard error
SOTA: State-of-the-art
SOV: The fractional overlap of segments
TM: Transmembrane
TN: True negatives
TP: True positive
