# Supplementary material for: Assessing the role of evolutionary information for enhancing protein language model embeddings
Source: Sci Rep. 2024 Sep 5;14:20692. doi: 10.1038/s41598-024-71783-8 (PMC11377704; doi:10.1038/s41598-024-71783-8)
Supplement: Supplementary file 1 — Supplementary Information. [file 41598_2024_71783_MOESM1_ESM.pdf]

Supporting Online Material for: Assessing the Role of Evolutionary Information for Enhancing Protein Language Model Embeddings

Kyra Erckert & Burkhard Rost

Table of Contents for SOM

TABLE OF CONTENTS FOR SOM 1

SHORT DESCRIPTION OF SUPPORTING ONLINE MATERIAL 2

1. ADDITIONAL RESULTS 2
  1.1 Secondary Structure Prediction 2
    1.1.1 Test Set 2
      Table S1: Q_3 and SOV performances on test set for different models. 2
      Table S2: MCC performances on test set for different models. 3
    1.1.2. Validation Set 5
      Figure S1: Q_3 performances of different model and embedding types on validation set. 5
      Table S3: Q_3 and SOV performances on validation set for different models. 6
      Table S4: MCC performances on validation set for different models. 7
    1.1.3 Weight Visualizations 8
      Figure S2: Visualization of CNN layer weights in the first layer of PSSMConcat 8
      Figure S3: Visualization of CNN layer weights in the concatenation layer of PSSMSplit 9
  1.2 Conservation Prediction 10
      Figure S4: Trade-off of conservation thresholds in terms of MCC and Q_2 10
      Figure S5: Raw embeddings vs MSACons for conservation prediction 11

2. DISCUSSION 12
  2.1 Limitations of Family Size Analysis 12
  2.2 Limitations of Training pLMs without Evolutionary Information 12
  2.3 Limitations of biasing pLM training towards Families 13

3. MATERIALS AND METHODS 14
  3.1 ML Architectures 14
      Figure S6: Sketch of the architecture used for raw embeddings, MSA embeddings and MSAConsensus 14
      Figure S7: Sketch of PSSMSplit 15
      Figure S8: Sketch of PSSMConcat 16
  3.2 MSAConsensus predictions for SETH, VESPA, bindEmbed21DL and TMbed 17
  3.3 Additional Performance Measures 17

4. RELATED WORK 18
      Table S5: Conservation MCC prediction performances. 18
      Table S6: Disorder spearman correlation. 18
      Table S7: Binding residue F_1 prediction performances. 19
      Table S8: Transmembrane segment Q_{ok} prediction performances. 20
      Table S9: Secondary Structure Q_3 prediction performance. 21
      Table S10: 3D protein structure prediction TM-scores. 21
      Table S11: SAV effect MCC prediction performances. 22
      Table S12: SAV effect prediction spearman correlation. 22
      Table S13: CATH superfamily MCC prediction performances. 23
      Table S14: SCOP prediction spearman correlation. 24
      Table S15: Localization Q_{10} prediction performances. 24

Table S16: GO function prediction F_{max} performance. 25
REFERENCES FOR SUPPORTING ONLINE MATERIAL 26

Short Description of Supporting Online Material

In this document, we provide additional tables and figures in context of our work. In the Additional Results section, we include tables with the exact performances for our models using SeqVec[1], ProtBert[2] and ProtT5[2] embeddings on the test and validation set (Table S1-S4) and a bar plot with the Q_3 performance on the validation set (Fig. S1). We also provide visualizations of the learned weights of our PSSMSplit and PSSMConcat models. We provide additional results on conservation predictions, including a line plot showing Q2 and MCC performance for different conservation cutoffs based on MMseqs2[3] alignments (Fig. S2) and a bar plot on the performance of different conservation prediction methods (Fig. S3). In the Discussion section, we included further details on the limitations discussed in the main text. These have been omitted from the main text to keep the manuscript concise but may still be of interest to some readers. In the Materials and Methods section, we provide visualizations of our network architectures (Fig. S4-6) and further explanations of MSAConsensus predictions and additional performance measures that have been used for method comparisons. In the Related Work section, we provide tables with the exact performances of the methods shown in the main text in Fig. 2 and 3 as well as additional methods for the same prediction task (Table S5-16).

1. Additional Results

1.1 Secondary Structure Prediction

1.1.1 Test Set

Table S1: Q_3 and SOV performances on test set for different models.

pLM	Model	Q_3	SOV
**SeqVec**	Raw embeddings	71.6 ± 0.5	58.9 ± 0.7
	MSA embeddings	**77.7 ± 0.6**	**66.9 ± 0.7**
	MSAConsensus	75.9 ± 0.4	64.8 ± 0.8
	PSSMConcat	75.2 ± 0.4	64.2 ± 0.7
	PSSMSplit	74.5 ± 0.5	62.6 ± 0.7
**ProtBert**	Raw embeddings	80.9 ± 0.4	70.7 ± 0.9
	MSA embeddings	**81.7 ± 0.4**	**71.6 ± 0.8**
	MSAConsensus	80.8 ± 0.4	69.5 ± 0.8

pLM	Model	Q_3	SOV
	PSSMConcat	80.5 ± 0.4	71.0 ± 0.8
	PSSMSplit	80.9 ± 0.4	71.3 ± 0.8
**ProtT5**	Raw embeddings	**84.2 ± 0.4**	**75.1 ± 0.8**
	MSA embeddings	83.8 ± 0.4	74.5 ± 0.8
	MSAConsensus	83.4 ± 0.4	73.1 ± 0.8
	PSSMConcat	83.6 ± 0.4	74.2 ± 0.8
	PSSMSplit	83.7 ± 0.4	74.7 ± 0.8
**None**	Majority class baseline	43.0 ± 0.6	0
	Distribution baseline	36.1 ± 0.2	16.5 ± 0.3

Q_3 and SOV performance on the TEST100 dataset are shown. For SeqVec models, the MSA embeddings clearly outperform all other models in both performance measures. For ProtBert models, a significantly improvement can still be observed for Q_3 performance of MSA embeddings but for the SOV only a numerical difference. For ProtT5 models, raw embeddings perform similar to MSA embeddings with raw embeddings achieving the numerically highest performance. All models clearly outperform the Majority class and Distribution baseline. ± values mark the standard error (Eqn. 7). For each column, numerically highest performances for SeqVec, ProtBert and ProtT5 are highlighted in bold.

Distribution baseline: randomly predict labels by drawing from given class distribution.

Majority class baseline: predict majority class.

Table S2: MCC performances on test set for different models.

pLM	Model	MCC_H	MCC_E	MCC_-
**SeqVec**	Raw embeddings	0.56 ± 0.01	0.49 ± 0.01	0.501 ± 0.007
	MSA embeddings	**0.68 ± 0.01**	**0.60 ± 0.01**	**0.591 ± 0.006**
	MSAConsensus	0.65 ± 0.01	0.57 ± 0.01	0.585 ± 0.007
	PSSMConcat	0.63 ± 0.01	0.55 ± 0.01	0.549 ± 0.006
	PSSMSplit	0.62 ± 0.01	0.53 ± 0.01	0.543 ± 0.006
**ProtBert**	Raw embeddings	0.73 ± 0.01	0.65 ± 0.01	0.636 ± 0.007
	MSA embeddings	**0.74 ± 0.01**	**0.67 ± 0.01**	**0.645 ± 0.006**
	MSAConsensus	0.72 ± 0.01	0.66 ± 0.01	0.637 ± 0.006
	PSSMConcat	0.73 ± 0.01	0.65 ± 0.01	0.626 ± 0.007
	PSSMSplit	0.73 ± 0.01	0.64 ± 0.01	0.631 ± 0.007
**ProtT5**	Raw embeddings	**0.78 ± 0.01**	**0.72 ± 0.01**	**0.687 ± 0.007**
	MSA embeddings	0.77 ± 0.01	0.71 ± 0.01	0.679 ± 0.007

pLM	Model	MCC_H	MCC_E	MCC_-
	MSAConsensus	0.75 ± 0.01	**0.72 ± 0.01**	0.674 ± 0.007
	PSSMConcat	**0.78 ± 0.01**	0.71 ± 0.01	0.676 ± 0.007
	PSSMSplit	0.77 ± 0.01	0.71 ± 0.01	0.679 ± 0.007
**None**	Majority class baseline	0	0	0
	Distribution baseline	0.001 ± 0.004	0.004 ± 0.004	0.005 ± 0.004

MCC performances on the TEST100 dataset are shown. For SeqVec models, the MSA embeddings clearly outperform all other models in all classes. For ProtBert models, a significantly improvement can still be observed for using MSA embeddings in comparison to raw embeddings for sheet predictions. For the helix and other class raw embeddings and MSA embeddings perform similar. For ProtT5 models, all models perform similar but raw embeddings achieving the numerically highest performance in all 3 classes. ± values mark the standard error (Eqn. 7). For each column, numerically highest performances for SeqVec, ProtBert and ProtT5 are highlighted in bold.

Distribution baseline: randomly predict labels by drawing from given class distribution.

Majority class baseline: predict majority class.

1.1.2. Validation Set

Figure S1: Q_3 performances of different model and embedding types on validation set.

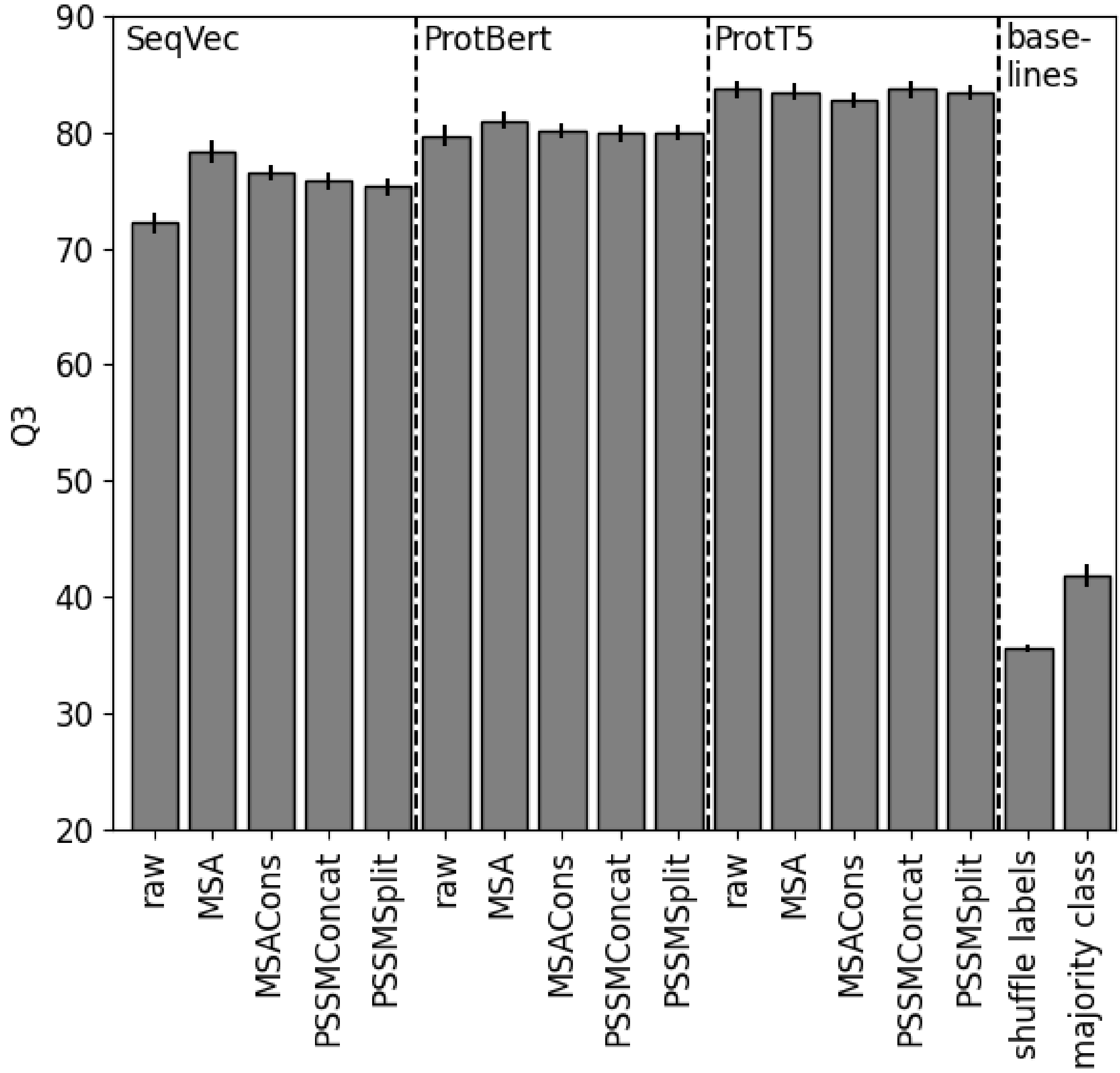

Average Q3 performance (y-axis) and confidence intervals (wicks) on the validation set for model and embedding combinations (x-axis). For embedding creation, SeqVec, ProtBert and ProtT5 were used. Raw embeddings refers to unaltered embeddings from pLMS, MSA embeddings are embeddings enriched with evolutionary information, MSACons refers to predictions obtained by averaging over predictions according to an MSA for each query sequence and PSSMConcat and PSSMSplit refers to model using raw embeddings and PSSMs as additional input features. MSA embeddings result in a significant performance boost for ProtBert and SeqVec embeddings but do not improve performance for ProtT5. shuffle labels baseline: randomly predict labels by drawing from given class distribution. majority class baseline: predict majority class.

Table S3: Q_3 and SOV performances on validation set for different models.

pLM	Model	Q3	SOV
**SeqVec**	Raw embeddings	72.2 ± 0.5	59.2 ± 0.7
	MSA embeddings	**78.3 ± 0.6**	**67.2 ± 0.7**
	MSAConsensus	76.5 ± 0.4	65.1 ± 0.8
	PSSMConcat	75.8 ± 0.4	64.8 ± 0.7
	PSSMSplit	75.3 ± 0.4	63.5 ± 0.7
**ProtBert**	Raw embeddings	79.7 ± 0.5	69.1 ± 0.8
	MSA embeddings	**81.0 ± 0.4**	**70.8 ± 0.7**
	MSAConsensus	80.1 ± 0.4	68.9 ± 0.8
	PSSMConcat	79.9 ± 0.4	69.9 ± 0.8
	PSSMSplit	80.0 ± 0.4	70.0 ± 0.7
**ProtT5**	Raw embeddings	**83.7 ± 0.4**	**74.7 ± 0.7**
	MSA embeddings	83.5 ± 0.4	73.8 ± 0.7
	MSAConsensus	82.8 ± 0.4	72.6 ± 0.7
	PSSMConcat	**83.7 ± 0.4**	74.6 ± 0.7
	PSSMSplit	83.4 ± 0.4	74.4 ± 0.7
**None**	Majority class baseline	41.8 ± 0.6	0
	Distribution baseline	35.6 ± 0.2	16.5 ± 0.3

Q_3 and SOV performance on the VAL100 dataset are shown. For SeqVec models, the MSA embeddings clearly outperform all other models in both performance measures. For ProtBert models, a significantly improvement can still be observed for Q_3 and SOV performance of MSA embeddings in comparison to raw embedding but the difference is smaller than the one observed for SeqVec. For ProtT5 models, raw embeddings perform similar to MSA embeddings with raw embeddings achieving the numerically highest performance. All models clearly outperform the Majority class and Distribution baseline. ± values mark the standard error (Eqn. 7). For each column, numerically highest performances for SeqVec, ProtBert and ProtT5 are highlighted in bold.

Distribution baseline: randomly predict labels by drawing from given class distribution.

Majority class baseline: predict majority class.

Table S4: MCC performances on validation set for different models.

pLM	Model	MCC_H	MCC_E	MCC_-
**SeqVec**	Raw embeddings	0.56 ± 0.01	0.506 ± 0.015	0.502 ± 0.006
	MSA embeddings	**0.69 ± 0.01**	**0.61 ± 0.01**	**0.613 ± 0.006**
	MSAConsensus	0.66 ± 0.01	0.58 ± 0.01	0.596 ± 0.007
	PSSMConcat	0.63 ± 0.01	0.56 ± 0.01	0.548 ± 0.006
	PSSMSplit	0.62 ± 0.01	0.54 ± 0.01	0.550 ± 0.006
**ProtBert**	Raw embeddings	0.69 ± 0.01	0.63 ± 0.01	0.613 ± 0.008
	MSA embeddings	**0.71 ± 0.01**	**0.66 ± 0.01**	**0.630 ± 0.006**
	MSAConsensus	0.69 ± 0.01	0.64 ± 0.01	0.621 ± 0.007
	PSSMConcat	0.697 ± 0.004	0.64 ± 0.01	0.614 ± 0.007
	PSSMSplit	0.70 ± 0.01	0.63 ± 0.01	0.613 ± 0.007
**ProtT5**	Raw embeddings	**0.76 ± 0.01**	**0.70 ± 0.01**	**0.679 ± 0.006**
	MSA embeddings	0.75 ± 0.01	**0.70 ± 0.01**	0.673 ± 0.006
	MSAConsensus	0.74 ± 0.01	0.69 ± 0.01	0.663 ± 0.006
	PSSMConcat	0.75 ± 0.01	**0.70 ± 0.01**	0.670 ± 0.006
	PSSMSplit	0.75 ± 0.01	0.69 ± 0.01	0.672 ± 0.006
**None**	Majority class baseline	0	0	0
	Distribution baseline	0.005 ± 000.4	0.003 ± 0.004	0.001 ± 0.003

MCC performances on the VAL100 dataset are shown. For SeqVec models, the MSA embeddings clearly outperform all other models in all classes. For ProtBert models, a significantly improvement can still be observed for using MSA embeddings in comparison to raw embeddings. For ProtT5 models, all models perform similar but raw embeddings achieving the numerically highest performance in all 3 classes. ± values mark the standard error (Eqn. 7). For each column, numerically highest performances for SeqVec, ProtBert and ProtT5 are highlighted in bold.

Distribution baseline: randomly predict labels by drawing from given class distribution.

Majority class baseline: predict majority class.

1.1.3 Weight Visualizations

Figure S2: Visualization of CNN layer weights in the first layer of PSSMConcat

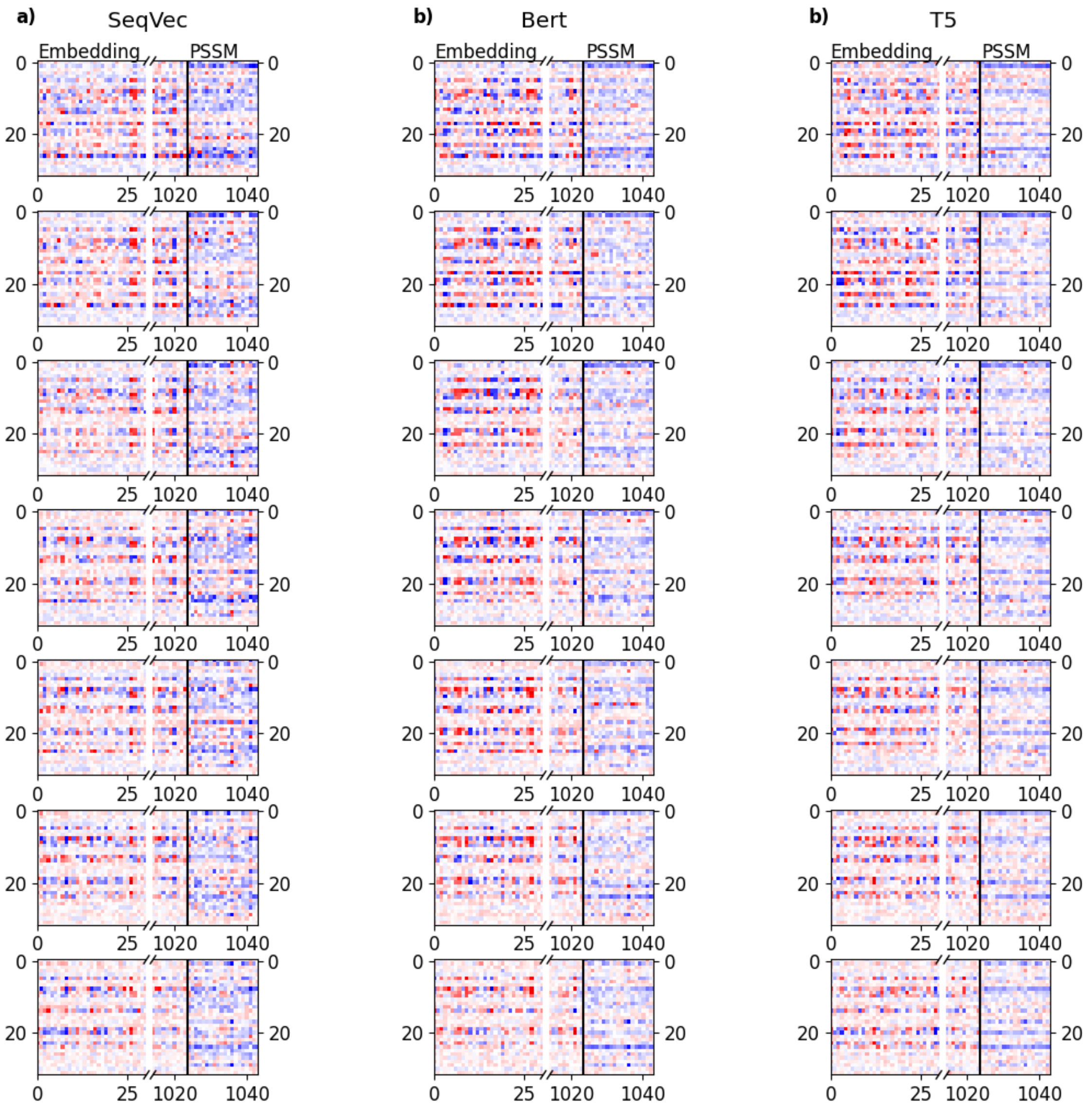

The heatmaps visualize the learned weights of the first layer of the PSSMConcat architecture, which integrates information from both embeddings and PSSMs. The weights for three different embedding types are shown: SeqVec (a), ProtBert (b), and ProtT5 (c). Each heatmap in a subplot corresponds to a different filter in the CNN layer. Weights are visualized using a divergent colour map centred around 0, where weights close to 0 are represented in white, positive values in red, and negative values in blue. For clarity, only the first 30 and last 10 embedding dimensions are visualized alongside the PSSM dimensions. The black lines indicate where embedding inputs end and the PSSM inputs start.

Figure S3: Visualization of CNN layer weights in the concatenation layer of PSSMSplit

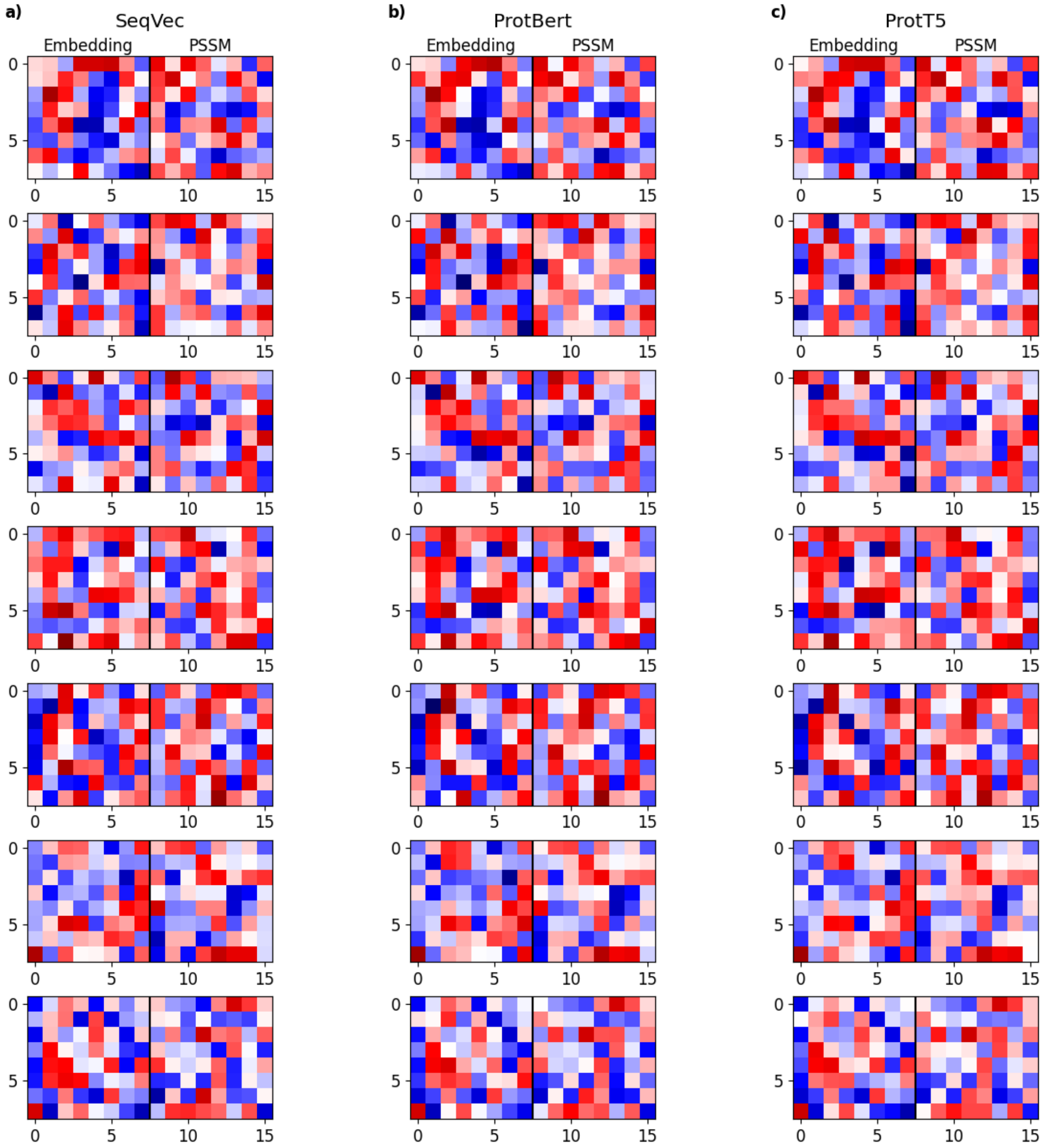

The heatmaps visualize the learned weights of the concatenation layer of the PSSMSplit architecture, which integrates extracted features from both embeddings and PSSMs. The weights for three different embedding types are shown: SeqVec (a), ProtBert (b), and ProtT5 (c). Each heatmap in a subplot corresponds to a different filter in the CNN layer. Weights are visualized using a divergent colour map centred around 0, where weights close to 0 are represented in white, positive values in red, and negative values in blue. The black lines indicate where embedding features end and the PSSM features start.

1.2 Conservation Prediction

We tested two sets of methods to compute residue conservation within protein families. The first set, including ConSeq[4] and MMseqs2[3], read conservation from the multiple sequence alignment (MSA) describing the family. The second set, including VESPA[5] and our newly introduced VESPA MSACons, predict conservation based on embeddings. Initially, the reported performance of the default embedding-only version of VESPA showed a stronger correlation with ConSeq than any alignment-informed version, trying to boost embeddings by explicitly using MSA information, that we tested (Fig. S5). For predicting per-residue conservation in a family (as derived from ConSurf[6]), we were surprised to find that integrating MSA-based information significantly decreased performance in comparison to the reported performance since the ground truth -conservation- is explicitly encoded into the MSA. However, we were unable to reproduce the reported performance when rerunning VESPA on the sequences of the ConSurf10k test set. Our re-evaluation showed that VESPA's performance was significantly lower, achieving similar performance to MSACons with MMseqs2 alignments. Furthermore, when using the original MAFFT alignments from ConSurfDB for our MSACons approach, we observed statistically significant (at 95% confidence interval – CI: ±1.96 stderr) performance improvements to our re-evaluation of VESPA (Fig. S5). Our re-evaluation suggests that the published model performance is actually much lower than reported, aligning closely with our MSACons approach using MMseqs2 MSAs, and even being surpassed when using MAFFT[7] MSAs. We hypothesize that this improvement is due to the consistency between the MSAs used for label generation and those used in our MSACons approach.

Figure S4: Trade-off of conservation thresholds in terms of MCC and Q_2

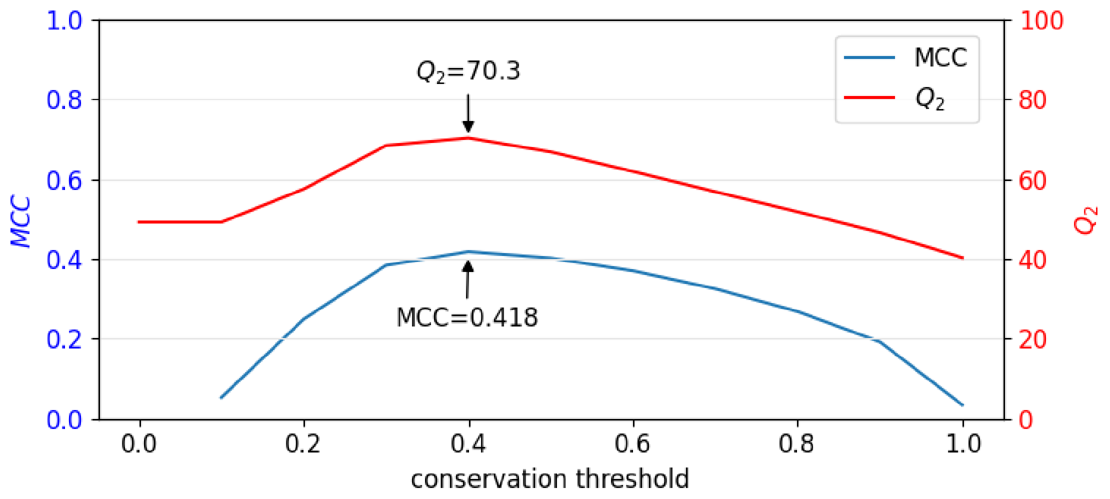

Q2 (right, red) and MCC (left, blue) performance (y-axis) at different conservation thresholds (x-axis) on the ConSurf10k dataset. The best performance (MCC=0.418, Q_2=70.3%) can be achieved with a conservation threshold of 0.4. A position in an MSA is considered conserved if at least the percentage given by the threshold of amino acids aligned at that position are the same.

Figure S5: Raw embeddings vs MSACons for conservation prediction

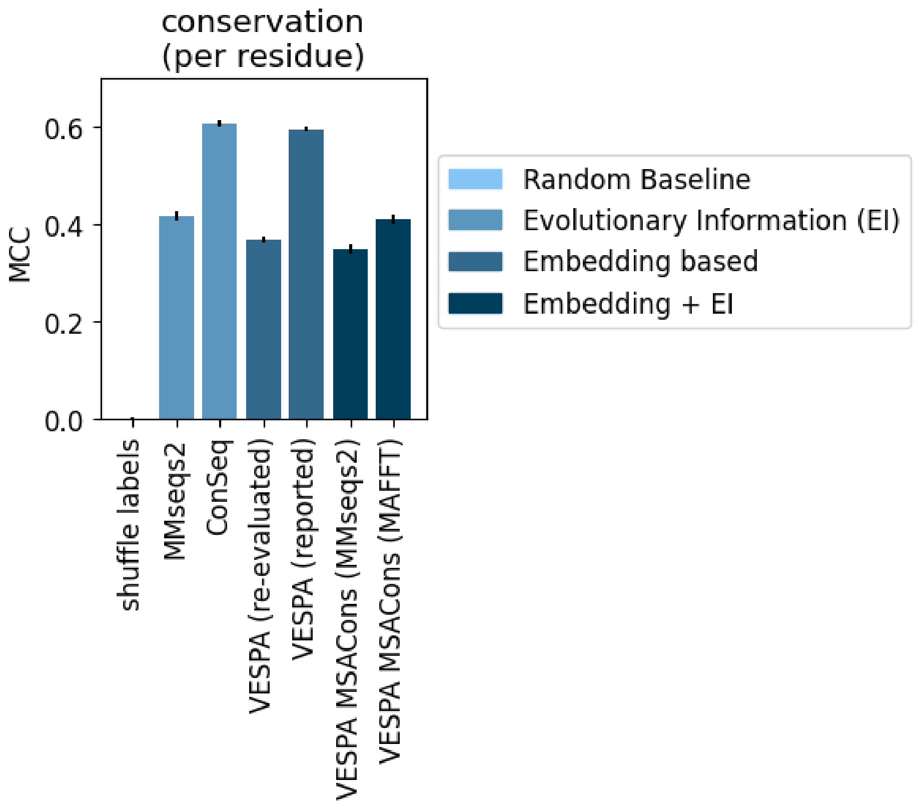

Explicitly adding embeddings through multiple sequence alignments (MSAs, here MSACons) is on-par (MMseqs2) or increases (MAFFT) performance for conservation prediction in comparison to our VESPA re-evaluation. Error bars mark 1.96% standard errors, i.e., the 95% confidence interval (CI). Shown are MCC performances on ConSurf10k test[5] set.
The original MSA-based methods were (left to right): MMseqs2[3], ConSeq[5]; the original embedding-based method: VESPA[5].

2. Discussion

2.1 Limitations of Family Size Analysis

A major obstacle in comprehensively establishing if proteins from small families (few proteins in MSA) are predicted differently from those in large families is two-fold. Firstly, very few high-resolution experiments target proteins from small families.[8] This implies that differences will likely not be statistically significant. Secondly and more importantly, smaller families are likely to differ in their biophysical, dynamical, and functional characteristics from larger families[9]. The simple argument of the theoretical biophysicist Alyosha Finkelstein for this assumption can be sketched as follows (using the term *fold* to loosely describe the main 3D scaffold conserved between two proteins with diverged sequences): not all folds are equally likely to realize and stabilize; thus, some folds are more likely to occur than others. In other words, these folds are *fitter*, which explains why some folds are more populated (larger families) than others. If true, we expect those from more prominent families to have different biophysical features than those from smaller families. Thus, even if we could collect enough samples in the future, these would still not be representative because we would compare apples and oranges.

2.2 Limitations of Training pLMs without Evolutionary Information

That removing all evolutionary information from training the pLMs is currently impossible can be concluded by a simple number game: explicitly using MSAs only failed for pLMs such as ProtT5[2] trained with over $2x10^9$ sequences from BFD[10]; the entire UniProt[11] with about ten times fewer sequences ($0.2x10^9$) did not suffice (ProtBert, Fig. 1). While a data reduction by a factor of ten already makes it impossible to narrow down what information pLMs cover, training pLMs only on non-redundant (e.g., at the level of less than 20% pairwise sequence identity) would reduce the data more likely by a factor of 100-1000 than ten. This means that before we have databases with 100-1000 times more sequences, this remains completely impossible. At the current rate of sequence databases by far outgrowing the fastest evolving key of modern growth, namely computer chips, this would still require 10-20 years.

Additionally, we know that pLMs such as ProtT5 resolve more frequently occurring amino acids more accurately than less frequent amino acids (M Heinzinger, TUM, unpublished and [5]). However, the frequency of most (Leucine ~10%) and least frequent amino acid (Cysteine ~1.5%) differ by less than a factor of ten. In contrast, finding two proteins from the same family in the ocean of unrelated pairs is a much less frequent event, as evident from the following back-of-an-envelope calculation: The most prominent families have about 100k (100,000) proteins[12] (as an average, this is a gross overestimate because the 100th largest family is already over 10 times smaller). Assume this to be the average for all families. When feeding BFD with $2x10^9$ sequences into pLMs, pairs from the same family would, on average, occur every 20,000th time ($2x10^9$ / 10^5), i.e., the difference between positives and negatives would be 2,000 times larger for the *same family/not* than for different types of amino acids. While this number argument is, strictly speaking, no proof, it still illustrates the magnitude of the problem.

2.3 Limitations of biasing pLM training towards Families

One idea for biasing pLM training towards families could be to systematically pick all family members in one batch, i.e., by not selecting the next protein to train randomly. This proposition exceeds our computing resources because we simply have no funding for retraining a foundation pLM, in particular, given the limited chance of success. Furthermore, it remains unclear how informative a negative result would be: interfering with random choices on such an extreme level might do much damage, as we learned when first trying to use a simple neural network for secondary structure prediction and choosing samples one protein at a time, rather than randomly[13]. Thus, if such an approach wouldn't improve, the lack of improvement might be attributed to "incorrect sampling," evading the answer to our question correlation or capture once again. Conversely, if biased family sampling improved, we would gain insights. However, we hold this so unlikely that we would hesitate to invest substantial resources toward this end even if we had them.

3. Materials and Methods

3.1 ML Architectures

Figure S6: Sketch of the architecture used for raw embeddings, MSA embeddings and MSAConsensus

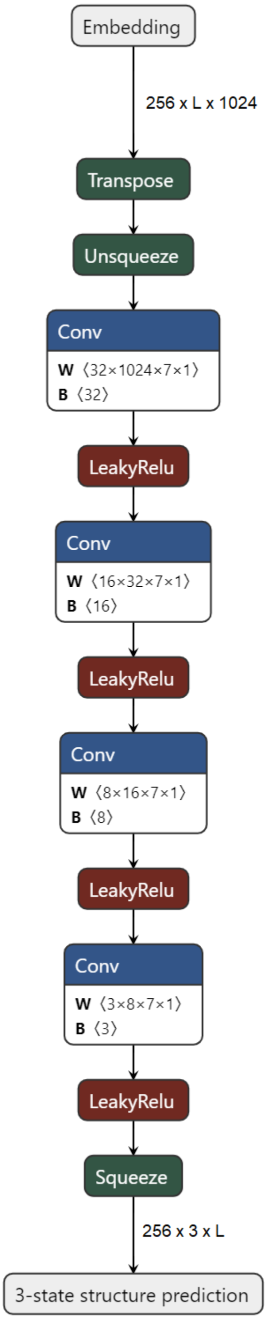

Architecture of the raw embeddings, MSA embeddings and MSAConsensus models. Sequences are processed in batches of 256 and are dynamically padded to the length of the longest sequence in a batch (L). The 1024 dimensional embeddings are processed by 4 convolutional layers before outputting a prediction in 3 classes (beta-strand, alpha helix or other).

Figure S7: Sketch of PSSMSplit

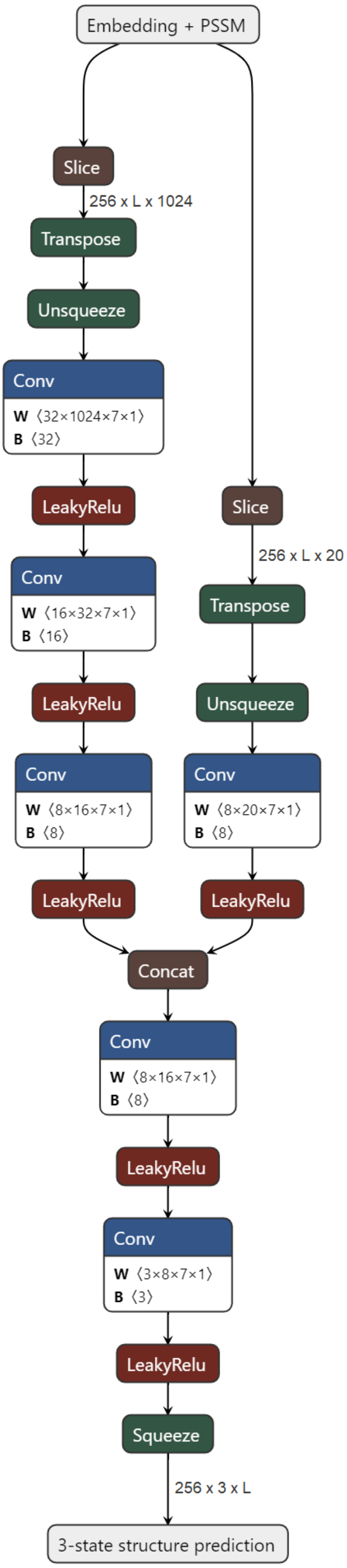

Model architecture of the PSSMSplit model. Sequences are processed in batches of 256 and are dynamically padded to the length of the longest sequence in a batch (L). The 1024 dimensional embeddings are processed by 3 consecutive convolutional layers (left side) and the PSSMs are processed by a single convolutional layer (right side). The outputs of both are concatenated before being further processed by 2 additional convolutional layers before outputting a prediction in 3 classes (beta-strand, alpha helix or other).

Figure S8: Sketch of PSSMConcat

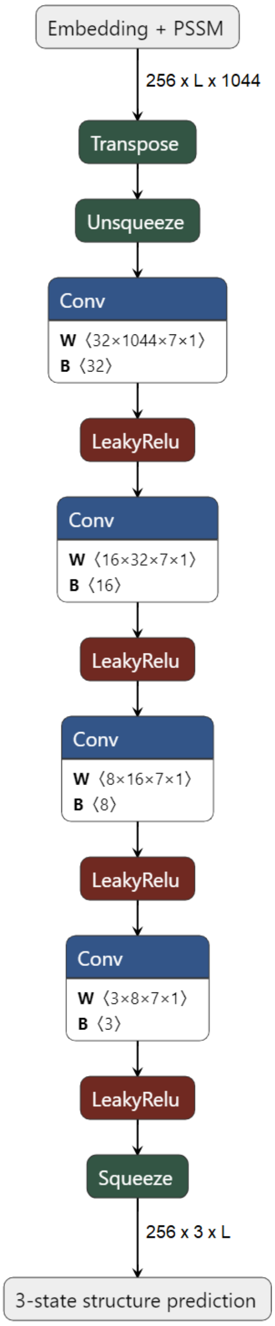

Model architecture of the PSSMConcat model. Sequences are processed in batches of 256 and are dynamically padded to the length of the longest sequence in a batch (L). The 1024 dimensional embeddings are concatenated with the 20 dimensional PSSM, resulting in a 1044 dimensional input for each residue. The input is processed by 4 convolutional layers before outputting a prediction in 3 classes (beta-strand, alpha helix or other).

3.2 MSAConsensus predictions for SETH, VESPA, bindEmbed21DL and TMbed

Identical to the MSAConsensus predictions for our own method, we created MSAs with MMseqs2[3] for all relevant test sets (ConSurf10k[5] for VESPA[5], TestSet225[14] for bindEmbed21DL[14], 57 β-TMP[15] and 571 α-TMPs[15] for TMbed[15], CheZOD117[16] for SETH[17]). We used the method of interest to generate predictions for each sequence in the MSA. The per-residue predictions for each aligned sequence were mapped to the aligned position of the residue in the query sequence. For SETH, the mean of all predictions, that map to an individual query residue was computed and used as the MSAConsensus prediction. For bindEmbed21 a majority vote out of 8 classes (non-binding, metal, nuclear, small, metal and nuclear, metal and small, nuclear and small, binding all 3), for TMbed a majority vote out of 2 classes (either TM-helix/non helix or TM-sheet/non sheet) and for VESPA a majority vote out of 9 classes (1-9) was used to determine the MSAConsensus prediction.

3.3 Additional Performance Measures

For secondary structure prediction, besides Q_3, we evaluated performance through several additional measures. For simplicity, we used the following standard annotations, with x ∊ {H, E, -}: True positives (TP_x) were correctly predicted secondary structures of class x. In contrast, false positives (FP_x) were predicted secondary structures of class x that were experimentally annotated as not x. True negatives (TN_x) were correctly predicted as not of class x, and false negatives (FN_x) were residues annotated as class x but incorrectly predicted to be not x. We calculated MCC (Eqn. 1) separately for all secondary structure classes:

$$MCC_x = \frac{TP_x \times TN_x - FN_x \times FP_x}{\sqrt{(TP_x+FP_x)\times(TP_x+FN_x)\times(TN_x+FP_x)\times(TN_x+FN_x)}} \quad \text{(Eqn. S1)}$$

As additional combined performance measures, we used the fraction overlap measure (SOV, Eqn. 2[18-20]):

$$SOV = \frac{1}{\sum_{i=1}^{N_C} N(i)} \sum_{i=1}^{N_C} \sum_{S(i)} \left[\frac{minov(s_0,s_1) + \delta(s_0,s_1)}{maxov(s_0,s_1)} len(s_0)\right] \quad \text{(Eqn. S2)}$$

s_0 refers to all observed helix, strand, and other segments, and s_1 to all predicted segments. S(i) denotes the set of all overlapping pairs (s_0, s_1), and N(i) is the sum of elements in S(i) and the number of all segments s_0 that are not overlapped by a predicted segment of identical state. The length of any segment s_0 in amino acid residues is given by len(s_0), the length of the actual overlap of a given state by minov(s_0, s_1), and the extend, for which at least one residue is in the given state by maxov(s_0, s_1). δ(s_0, s_1) is defined as:

$$\delta(s_0, s_1) = \frac{3}{\sum_{j=1}^{N_S} (\frac{len(s_j)}{len(s_r)})^2} \times \frac{len(s_0)}{len(s_r)} \times \frac{minov(s_0,s_1)}{maxov(s_0,s_1)} \quad \text{(Eqn. S3)}$$

4. Related Work

Table S5: Conservation MCC prediction performances.

Method	Embedding based?	MCC	Dataset
ConSeq[5] (MMseqs2[3], PSI-BLAST[21])	X	**0.608 ± 0.006**[5]	ConSurf10k test[5]
MMseqs2[3]	X	0.418 ± 0.009	ConSurf10k test[5]
VESPA[5]	✓	**0.596 ± 0.006**[5]	ConSurf10k test[5]
VESPA[5] re-evaluated	✓	0.37 ± 0.01	ConSurf10k test[5]
VESPA[5] (MSAConsensus, MMseqs2[3])	✓	0.35 ± 0.01	ConSurf10k test[5]
VESPA[5] (MSAConsensus, MAFFT[7])	✓	0.41 ± 0.01	ConSurf10k test[5]
Random Rate[5]	X	0.000 ± 0.006[5]	ConSurf10k test[5]

MCC performance of ConSeq[5], MMseqs2[3], VESPA[5], our own evaluation of VESPA, VESPA[5] using MSAConsensus predictions (with MMseqs2 and MAFFT MSAs) and Random Rate baseline[5] for conservation prediction on the ConSurf10k test set[5]. For conservation prediction, the embedding based VESPA and the alignment-based ConSeq are reported to achieve similar performances. The MSAConsensus prediction with MMseqs2 MSAs of VESPA achieves a similar performance to our re-evaluation of VESPA and outperforms it if MAFFT MSAs are used. Performances for VESPA, ConSeq and Random Rate were obtained from Marquet et al.. For the MCC column significantly best results are highlighted in bold.

Random Rate: randomly predict labels by drawing from given class distribution.

Table S6: Disorder spearman correlation.

Method	Embedding based?	Spearman correlation	Dataset
ODiNPred[16]	X	0.67 ± 0.01[17]	CheZOD117[16]
SPOT-Disorder[22]	X	0.64[17]	CheZOD117[16]
SETH[17]	✓	**0.72 ± 0.01**[17]	CheZOD117[16]
SETH[17] (MSAConsensus)	✓	0.43 ± 0.05	CheZOD117[16]

**Method**	**Embedding based?**	**Spearman correlation**	**Dataset**
ADOPT(Esm1b)[23]	✓	0.69[17]	CheZOD117[16]
Random Rate	X	0.00 ± 0.01	CheZOD117[16]

Spearman correlation for the predictions of ODiNPred[16], SPOT-Disorder[22], SETH[17], SETH[17] using MSAConsensus predictions and ADOPT (Esm1b)[23] on disorder prediction on the CheZOD117 dataset[16]. For disorder prediction, the embedding-based method SETH clearly outperforms evolutionary information-based ones like ODiNPred and SPOT-Disorder. Performances for ODiNPred, SPOT-Disorder, SETH and ADOPT(ESM1b) were obtained from Illzhöfer et al.. For the Spearman correlation column significantly best results are highlighted in bold.

Random Rate: randomly predict labels by drawing from given class distribution.

Table S7: Binding residue F_1 prediction performances.

**Method**	**Embedding based?**	**F_1**	**Dataset**
bindPredictML17[24]	X	34 ± 2[14]	TestSet225[14]
bindEmbed21DL[14]	✓	**47 ± 2**[14]	TestSet225[14]
bindEmbed21DL[14] (MSAConsensus)	✓	**50 ± 2**	TestSet225[14]
Random Rate	X	3.7	TestSet225[14]
ZeroR	X	0	TestSet225 [14]

F_1 performances of bindPredictML17[24], bindEmbed21DL[14], bindEmbed21DL[14] using MSAConsensus predictions, Random rate baseline and ZeroR baseline for binding residue prediction on the TestSet225[14]. For binding residue prediction the embedding based methods bindEmbed21DL (original as well as MSAConsensus predictions) significantly outperforms all other methods. All methods clearly outperform the Random Rate and ZeroR baselines. Performances for bindPredictML17 and bindEmbed21DL were obtained from Littmann et al. MSAConsensus predictions, Random Rate and ZeroR baselines were computed in the context of this work. For the F_1 column significantly best results are highlighted in bold.

Random Rate: randomly predict labels by drawing from given class distribution.

ZeroR: predict majority class.

Table S8: Transmembrane segment Q_{ok} prediction performances.

**Task**	**Method**	**Embedding based?**	**Q_{ok}**	**Dataset**
Beta barrel trans-membrane segment	BOCTOPUS2[25]	✗	**56.6 ± 18.9[15]**	57 β-TMP[15]
	TMbed[15]	✓	**80.5 ± 11.4[15]**	57 β-TMP[15]
	TMbed[15] (MSACons)	✓	**82.4 ± 10.1**	57 β-TMP[15]
	Random Rate	✗	0	57 β-TMP[15]
Alpha helix bundle transmembrane segment	TOPCONS2[26]	✗	41.0 ± 3.1[15]	571 α-TMPs[15]
	TMbed[15]	✓	62.4 ± 3.7[15]	571 α-TMPs[15]
	TMbed[15] (MSACons)	✓	**70.1 ± 3.7**	571 α-TMPs[15]
	Random Rate	✗	0	571 α-TMPs[15]

Q_{ok} of BOCTOPUS2[25], TMbed[15], TMbed[15] using MSAConsensus predictions and ZeroR baseline for beta barrel transmembrane segment prediction on the 57 β-TMP dataset[15] and TOPCONS2[26], TMbed[15], TMbed[15] using MSAConsensus predictions and ZeroR baseline for alpha helix bundle transmembrane segment prediction on the 571 α-TMPs dataset[15]. For beta barrel prediction, a numerically higher performance can be observed for both versions of the embedding based TMbed and over BOCTOPUS2. For alpha bundel prediction, a significantly higher performance can be observed for the embedding based TMbed MSAConsensus approach over TOPCONS2 and TMbed. All methods clearly outperform the Random Rate and ZeroR baselines. Performances were obtained from Bernhofer and Rost (2022) for beta barrel and alpha helix bundle transmembrane segment performances of BOCTOPUS2, TMbed and TOPCONS2. MSAConsensus predictions and Random Rate baseline were computed in the context of this work. If available, significantly best results are highlighted in bold.

Random Rate: randomly predict labels by drawing from given class distribution.

Table S9: Secondary Structure Q_3 prediction performance.

**Method**	**Embedding based?**	**Q_3**	**Dataset**
NetSurfP-2.0[27]	X	**82.0 ± 1.6**[2]	CASP12[28]
Ankh[29]	✓	**83.8 ± 3**[29]	CASP12[28]
ProtT5-XL-U50[2]	✓	**81.4 ± 1.6**[2]	CASP12[28]
ProtT5 raw embeddings (this work)	✓	**80 ± 1.5**	CASP12[28]
NetSurfP-3.0[30]	✓	79.1[30]	CASP12[28]
Random Rate	X	38.6	CASP12[28]
ZeroR	X	48.6	CASP12[28]

Q_3 performance of NetSurfP-2.0[27], Ankh[29], ProtT5-XL-U50[2], NetSurfP-3.0[30], the T5 raw embedding model from this work, Random Rate baseline and ZeroR baseline for secondary structure prediction in 3 classes on the CASP12 dataset [28]. Embedding based methods like our ProtT5 raw embeddings method, ProtT5-XL-U50 and Ankh, are able to compete with evolutionary information-based ones like NetSurfP-2.0. All methods clearly outperform the Random Rate and ZeroR baselines. Performances for NetSurfP-2.0, NetSurfP-3.0, Ankh, and ProtT5-XL-U50 were obtained from Elnaggar et al. and Hoie et al.. Random Rate and ZeroR baselines for secondary structure were computed in the context of this work. For the Q_3 column, significantly best results are highlighted in bold.

Random Rate: randomly predict labels by drawing from given class distribution.

ZeroR: predict majority class.

Table S10: 3D protein structure prediction TM-scores.

**Task**	**Method**	**Embedding based?**	**TM-score**	**Dataset**
3D protein structure	AlphaFold2[31]	X	0.88[32]	CAMEO[33]
	ESMFold[32]	✓	0.83[32]	CAMEO[33]
	AlphaFold2[31]	X	0.85[32]	CASP14[34]

Task	Method	Embedding based?	TM-score	Dataset
	ESMFold[32]	✓	0.68[32]	CASP14[34]
	Random Pair[35]	X	0.17[35]	Structures in PDB

TM-score performances of AlphaFold2[31] and ESMFold[32] on 3D protein structure prediction on the CAMEO[33] and CASP14[34] datasets. The embedding based ESMFold, which uses evolutionary information in the form of MSAs during inference performs similar to AlphaFold2 on the CAMEO dataset but AlphaFold2 outperforms ESMFold on the CASP14 dataset. Performances were obtained from Lin et al..

Table S11: SAV effect MCC prediction performances.

Method	Embedding based?	MCC	Dataset
SignalP-5.0 original[36]	X	0.821[37]	SignalP-5.0 benchmark[36]
DEEPSIG[38]	X	0.792[37]	SignalP-5.0 benchmark[36]
SignalP-5.0 retrain[37]	X	0.774[37]	SignalP-5.0 benchmark[36]
SignalP-6.0[37]	✓	0.868[37]	SignalP-5.0 benchmark[36]
Random Rate	X	0.00116 ± 0.00008	SignalP-5.0 benchmark[36]

MCC performance of SignalP-6.0[37], SignalP-5.0[36] (original and retrained) DEEPSIG[38] and Random Rate on Eukarya signal peptide prediction on the SignalP-5.0 benchmark dataset[36]. The embedding based SignalP-6.0 and the original SignalP-5.0 achieve similar performances, with a at least numerical improvement for SignalP-6.0. Performances for SignalP-5.0, DEEPSIG, and SignalP-6.0 were obtained from Nallapareddy et al.

Random Rate: randomly predict labels by drawing from given class distribution.

Table S12: SAV effect prediction spearman correlation.

Method	Embedding based?	Spearman correlation	Dataset
GEMME[39]	X	0.53 ± 0.13[39]	DMS41[40]
DeepSequence[40]	X	0.499[41]	DMS41[40]
ESM-1v[41] (+further training)	✓	0.519[41]	DMS41[40]

Method	Embedding based?	Spearman correlation	Dataset
Random Rate	X	-0.00079 ± 0.00007	DMS41[40]
VESPA[5]	✓	0.51 ± 0.02[5]	DMS39[40]
VESPAl[5]	✓	0.47 ± 0.02[5]	DMS39[40]

Spearman correlation of DeepSequence[40], GEMME[39], ESM-1v[41] and Random Rate for SAV effect prediction on the DMS41 dataset[40], VESPA[5] and VESPAl[5] performance for SAV effect prediction on the DMS39 dataset[40]. For SAV effect prediction, embedding based methods can achieve performances that are similar to the ones achieved by methods like GEMME, that use evolutionary information. Performances for VESPA, VESPAl, ESM-1v, DeepSequence and GEMME were obtained from Meier et al., Laine et al. and Marquet et al.

Random Rate: randomly predict labels by drawing from given class distribution.

Table S13: CATH superfamily MCC prediction performances.

Method	Embedding based?	MCC	Dataset
BLAST + toolkit (version: 2.11.0)[42]	X	0.310 ± 0.006[43]	TOP 1773[43]
CATHe[43]	✓	**0.855 ± 0.004**[43]	TOP 1773[43]
Linear Regression + ProtT5[43]	✓	0.831 ± 0.004[43]	TOP 1773[43]
Random Rate	X	0.00003 ± 0.00080[43]	TOP 1773[43]

MCC performance of BLAST[42], CATHe [43], Linear Regression + ProtT5[43] and Random Rate baseline for CATH superfamily prediction on the TOP1773 dataset[43]. For the CATH superfamily prediction, the embedding based method performance is significantly higher than all other methods. Performances were obtained from Nallapareddy et al.,. For the MCC column significantly best results are highlighted in bold.

Random Rate: randomly predict labels by drawing from given class distribution.

Table S14: SCOP prediction spearman correlation.

Method	Embedding based?	Spearman correlation	Dataset
TMalign[44]	✗	0.37405[45]	ASTRAL 2.06 test[46,47]
MT-LSTM[45]	✓	0.70062[45]	ASTRAL 2.06 test[46,47]
Random Rate	✗	0.0008	ASTRAL 2.06 test[46,47]

Spearman correlation for SCOP prediction on the ASTRAL 2.06 test set[46,47]. For SCOP predictions, the embedding based method MT-LSTM achieves a numerically higher performance then TMalign. Performances for TMalign and MT-LSTM were obtained from Bepler and Berger (2021).

Random Rate: randomly predict labels by drawing from given class distribution.

Table S15: Localization Q_{10} prediction performances.

Method	Embedding based?	Q_{10}	Dataset
DeepLoc[48]	✗	78[2]	DeepLoc Test[48]
LA(ProtT5)[49]	✓	**86.0 ± 0.3**[49]	DeepLoc Test[48]
Ankh[29]	✓	83.2 ± 2[29]	DeepLoc Test[48]
ProtT5-XL-U50[2]	✓	81[2]	DeepLoc Test[48]
Random Rate	✗	19.2	DeepLoc Test[48]
ZeroR	✗	33	DeepLoc Test[48]

Q_{10} performances of DeepLoc[48], LA(ProtT5)[49], Ankh[29], ProtT5-XL-U50[2], Random Rate baseline and ZeroR baseline for localization prediction in 10 classes on the DeepLoc Test set[48].For the localization prediction, the embedding based LA(ProtT5) performance is significantly higher than all other methods. All methods clearly outperform the Random Rate and ZeroR baselines. Performances for DeepLoc, LA(ProtT5), Ankh, and ProtT5-XL-U50 were obtained from Elnaggar et al., and Stärk at al.. Random Rate and ZeroR baselines were computed in the context of this work. For the Q_{10} column, significantly best results are highlighted in bold.

Random Rate: randomly predict labels by drawing from given class distribution.

ZeroR: predict majority class.

Table S16: GO function prediction F_{max} performance.

Task	Method	Embedding based?	F_{max}	Dataset
GO function BPO	DomFun[50]	✗	49 ± 2[50]	CAFA3[51]
	GOLabeler[52]	✗	40 ± 1[51]	CAFA3[51]
	goPredSim[53]	✓	37 ± 2[53]	CAFA3[51]
	Random Rate	✗	~25	CAFA3[51]
GO function CCO	DomFun[50]	✗	60.2[50]	CAFA3[51]
	GOLabeler[52]	✗	61 ± 1[51]	CAFA3[51]
	goPredSim[53]	✓	57 ± 2[53]	CAFA3[51]
	Random Rate	✗	~55	CAFA3[51]
GO function MFO	DomFun[50]	✗	62 ± 3[50]	CAFA3[51]
	GOLabeler[52]	✗	62 ± 1[51]	CAFA3[51]
	goPredSim[53]	✓	50 ± 3[53]	CAFA3[51]
	Random Rate	✗	~31	CAFA3[51]

F_{max} performances of DomFun[50], GOLabler[52] and goPredSim[53] on GO function prediction (biological process, cellular component, molecular function) on the CAFA3 dataset[51]. For all 3 aspects of function prediction, the evolutionary information based methods outperform the embedding based goPredSim. Performances were obtained from Rojano et al., Zhou et al. and Littmann et al..

References for Supporting Online Material

1 Heinzinger, M. *et al.* Modeling aspects of the language of life through transfer-learning protein sequences. *BMC Bioinformatics* **20**, 723 (2019). https://doi.org:10.1186/s12859-019-3220-8

2 Elnaggar, A. *et al.* ProtTrans: Toward Understanding the Language of Life Through Self-Supervised Learning. *IEEE Transactions on Pattern Analysis and Machine Intelligence* **44**, 7112-7127 (2022). https://doi.org:10.1109/TPAMI.2021.3095381

3 Steinegger, M. & Söding, J. MMseqs2 enables sensitive protein sequence searching for the analysis of massive data sets. *Nature Biotechnology* **35**, 1026-1028 (2017). https://doi.org:10.1038/nbt.3988

4 Berezin, C. *et al.* ConSeq: the identification of functionally and structurally important residues in protein sequences. *Bioinformatics (Oxford, England)* **20**, 1322-1324 (2004). https://doi.org:10.1093/bioinformatics/bth070

5 Marquet, C. *et al.* Embeddings from protein language models predict conservation and variant effects. *Human Genetics* **141**, 1629-1647 (2022). https://doi.org:10.1007/s00439-021-02411-y

6 Yariv, B. *et al.* Using evolutionary data to make sense of macromolecules with a “face-lifted” ConSurf. *Protein Science* **32**, e4582 (2023). https://doi.org:https://doi.org/10.1002/pro.4582

7 Katoh, K., Rozewicki, J. & Yamada, K. D. MAFFT online service: multiple sequence alignment, interactive sequence choice and visualization. *Briefings in Bioinformatics* **20**, 1160-1166 (2019). https://doi.org:10.1093/bib/bbx108

8 Liu, J., Montelione, G. T. & Rost, B. Novel leverage of structural genomics. *Nature Biotechnology* **25**, 849-851 (2007).

9 Finkelstein, A. V., Badretdinov, A. Y. & Gutin, A. M. Why do protein architectures have Boltzmann-like statistics? *Proteins: Structure, Function, and Genetics* **23**, 142-150 (1995).

10 Steinegger, M., Mirdita, M. & Soding, J. Protein-level assembly increases protein sequence recovery from metagenomic samples manyfold. *Nat Methods* **16**, 603-606 (2019). https://doi.org:10.1038/s41592-019-0437-4

11 The UniProt Consortium. UniProt: the universal protein knowledgebase in 2021. *Nucleic Acids Res* **49**, D480-D489 (2021). https://doi.org:10.1093/nar/gkaa1100

12 Dawson, N. L., Sillitoe, I., Lees, J. G., Lam, S. D. & Orengo, C. A. CATH-Gene3D: Generation of the Resource and Its Use in Obtaining Structural and Functional Annotations for Protein Sequences. *Methods Mol Biol* **1558**, 79-110 (2017). https://doi.org:10.1007/978-1-4939-6783-4_4

13 Rost, B. *Neural networks and evolution - advanced prediction of protein secondary structure*, Dep. of Physics and Astronomy, University of Heidelberg, F.R.G., (1993).

14 Littmann, M., Heinzinger, M., Dallago, C., Weissenow, K. & Rost, B. Protein embeddings and deep learning predict binding residues for various ligand

classes. *Scientific Reports* **11**, 23916 (2021). https://doi.org:10.1038/s41598-021-03431-4

15 Bernhofer, M. & Rost, B. TMbed: transmembrane proteins predicted through language model embeddings. *BMC Bioinformatics* **23**, 326 (2022). https://doi.org:10.1186/s12859-022-04873-x

16 Dass, R., Mulder, F. A. A. & Nielsen, J. T. ODiNPred: comprehensive prediction of protein order and disorder. *Scientific Reports* **10**, 14780 (2020). https://doi.org:10.1038/s41598-020-71716-1

17 Ilzhöfer, D., Heinzinger, M. & Rost, B. SETH predicts nuances of residue disorder from protein embeddings. *Frontiers in Bioinformatics* **2** (2022).

18 Zemla, A., Venclovas, Č., Fidelis, K. & Rost, B. A modified definition of Sov, a segment-based measure for protein secondary structure prediction assessment. *Proteins: Structure, Function, and Bioinformatics* **34**, 220-223 (1999). https://doi.org:https://doi.org/10.1002/(SICI)1097-0134(19990201)34:2<220::AID-PROT7>3.0.CO;2-K

19 Liu, T. & Wang, Z. SOV_refine: A further refined definition of segment overlap score and its significance for protein structure similarity. *Source Code for Biology and Medicine* **13**, 1 (2018). https://doi.org:10.1186/s13029-018-0068-7

20 Rost, B., Sander, C. & Schneider, R. Redefining the goals of protein secondary structure prediction. *Journal of Molecular Biology* **235**, 13-26 (1994). https://doi.org:https://doi.org/10.1016/S0022-2836(05)80007-5

21 Madeira, F. *et al.* Search and sequence analysis tools services from EMBL-EBI in 2022. *Nucleic acids research*, gkac240 (2022). https://doi.org:10.1093/nar/gkac240

22 Hanson, J., Yang, Y., Paliwal, K. & Zhou, Y. Improving protein disorder prediction by deep bidirectional long short-term memory recurrent neural networks. *Bioinformatics* **33**, 685-692 (2017). https://doi.org:10.1093/bioinformatics/btw678

23 Redl, I. *et al.* ADOPT: intrinsic protein disorder prediction through deep bidirectional transformers. *bioRxiv*, 2022.2005.2025.493416 (2023). https://doi.org:10.1101/2022.05.25.493416

24 Schelling, M., Hopf, T. A. & Rost, B. Evolutionary couplings and sequence variation effect predict protein binding sites. *Proteins: Structure, Function, and Bioinformatics* **86**, 1064-1074 (2018). https://doi.org:https://doi.org/10.1002/prot.25585

25 Hayat, S., Peters, C., Shu, N., Tsirigos, K. D. & Elofsson, A. Inclusion of dyad-repeat pattern improves topology prediction of transmembrane β-barrel proteins. *Bioinformatics* **32**, 1571-1573 (2016). https://doi.org:10.1093/bioinformatics/btw025

26 Tsirigos, K. D., Peters, C., Shu, N., Käll, L. & Elofsson, A. The TOPCONS web server for consensus prediction of membrane protein topology and signal peptides. *Nucleic Acids Res.* **43**, W401-W407 (2015). https://doi.org:10.1093/nar/gkv485

27 Klausen, M. S. *et al.* NetSurfP-2.0: Improved prediction of protein structural features by integrated deep learning. *Proteins: Structure, Function, and*

Bioinformatics **87**, 520-527 (2019). https://doi.org:https://doi.org/10.1002/prot.25674

28 Abriata, L. A., Tamò, G. E., Monastyrskyy, B., Kryshtafovych, A. & Dal Peraro, M. Assessment of hard target modeling in CASP12 reveals an emerging role of alignment-based contact prediction methods. *Proteins: Structure, Function, and Bioinformatics* **86**, 97-112 (2018). https://doi.org:https://doi.org/10.1002/prot.25423

29 Elnaggar, A. *et al.* Ankh: Optimized Protein Language Model Unlocks General-Purpose Modelling. (2023). https://doi.org:10.48550/ARXIV.2301.06568

30 Høie, M. H. *et al.* NetSurfP-3.0: accurate and fast prediction of protein structural features by protein language models and deep learning. *Nucleic Acids Res.* **50**, W510-W515 (2022). https://doi.org:10.1093/nar/gkac439

31 Jumper, J. *et al.* Highly accurate protein structure prediction with AlphaFold. *Nature* **596**, 583-589 (2021). https://doi.org:10.1038/s41586-021-03819-2

32 Lin, Z. *et al.* Evolutionary-scale prediction of atomic-level protein structure with a language model. *Science* **379**, 1123-1130 (2023). https://doi.org:10.1126/science.ade2574

33 Haas, J. *et al.* Continuous Automated Model EvaluatiOn (CAMEO) complementing the critical assessment of structure prediction in CASP12. *Proteins: Structure, Function, and Bioinformatics* **86**, 387-398 (2018). https://doi.org:https://doi.org/10.1002/prot.25431

34 Kryshtafovych, A., Schwede, T., Topf, M., Fidelis, K. & Moult, J. Critical assessment of methods of protein structure prediction (CASP)—Round XIV. *Proteins: Structure, Function, and Bioinformatics* **89**, 1607-1617 (2021). https://doi.org:https://doi.org/10.1002/prot.26237

35 Xu, J. & Zhang, Y. How significant is a protein structure similarity with TM-score = 0.5? *Bioinformatics* **26**, 889-895 (2010). https://doi.org:10.1093/bioinformatics/btq066

36 Almagro Armenteros, J. J. *et al.* SignalP 5.0 improves signal peptide predictions using deep neural networks. *Nature Biotechnology* **37**, 420-423 (2019). https://doi.org:10.1038/s41587-019-0036-z

37 Teufel, F. *et al.* SignalP 6.0 predicts all five types of signal peptides using protein language models. *Nature Biotechnology* **40**, 1023-1025 (2022). https://doi.org:10.1038/s41587-021-01156-3

38 Savojardo, C., Martelli, P. L., Fariselli, P. & Casadio, R. DeepSig: deep learning improves signal peptide detection in proteins. *Bioinformatics* **34**, 1690-1696 (2018). https://doi.org:10.1093/bioinformatics/btx818

39 Laine, E., Karami, Y. & Carbone, A. GEMME: A Simple and Fast Global Epistatic Model Predicting Mutational Effects. *Molecular Biology and Evolution* **36**, 2604-2619 (2019). https://doi.org:10.1093/molbev/msz179

40 Riesselman, A. J., Ingraham, J. B. & Marks, D. S. Deep generative models of genetic variation capture the effects of mutations. *Nature Methods* **15**, 816-822 (2018). https://doi.org:10.1038/s41592-018-0138-4

41 Meier, J. *et al.* Language models enable zero-shot prediction of the effects of mutations on protein function. *bioRxiv*, 2021.2007.2009.450648 (2021). https://doi.org:10.1101/2021.07.09.450648

42 Altschul, S. F., Gish, W., Miller, W., Myers, E. W. & Lipman, D. J. Basic local alignment search tool. *Journal of Molecular Biology* **215**, 403-410 (1990). https://doi.org:https://doi.org/10.1016/S0022-2836(05)80360-2

43 Nallapareddy, V. *et al.* CATHe: Detection of remote homologues for CATH superfamilies using embeddings from protein language models. *Bioinformatics*, btad029 (2023). https://doi.org:10.1093/bioinformatics/btad029

44 Zhang, Y. TM-align: a protein structure alignment algorithm based on the TM-score. *Nucleic Acids Res.* **33**, 2302-2309 (2005). https://doi.org:10.1093/nar/gki524

45 Bepler, T. & Berger, B. Learning the protein language: Evolution, structure, and function. *Cell Systems* **12**, 654-669.e653 (2021). https://doi.org:https://doi.org/10.1016/j.cels.2021.05.017

46 Fox, N. K., Brenner, S. E. & Chandonia, J.-M. SCOPe: Structural Classification of Proteins—extended, integrating SCOP and ASTRAL data and classification of new structures. *Nucleic Acids Res.* **42**, D304-D309 (2014). https://doi.org:10.1093/nar/gkt1240

47 Bepler, T. & Berger, B. Learning protein sequence embeddings using information from structure. (2019). https://doi.org:10.48550/ARXIV.1902.08661

48 Almagro Armenteros, J. J., Sønderby, C. K., Sønderby, S. K., Nielsen, H. & Winther, O. DeepLoc: prediction of protein subcellular localization using deep learning. *Bioinformatics* **33**, 3387-3395 (2017). https://doi.org:10.1093/bioinformatics/btx431

49 Stärk, H., Dallago, C., Heinzinger, M. & Rost, B. Light attention predicts protein location from the language of life. *Bioinformatics Advances* **1**, vbab035 (2021). https://doi.org:10.1093/bioadv/vbab035

50 Rojano, E. *et al.* Assigning protein function from domain-function associations using DomFun. *BMC Bioinformatics* **23**, 43 (2022). https://doi.org:10.1186/s12859-022-04565-6

51 Zhou, N. *et al.* The CAFA challenge reports improved protein function prediction and new functional annotations for hundreds of genes through experimental screens. *Genome Biology* **20**, 244 (2019). https://doi.org:10.1186/s13059-019-1835-8

52 You, R. *et al.* GOLabeler: improving sequence-based large-scale protein function prediction by learning to rank. *Bioinformatics* **34**, 2465-2473 (2018). https://doi.org:10.1093/bioinformatics/bty130

53 Littmann, M., Heinzinger, M., Dallago, C., Olenyi, T. & Rost, B. Embeddings from deep learning transfer GO annotations beyond homology. *Scientific Reports* **11**, 1160 (2021). https://doi.org:10.1038/s41598-020-80786-0
